# Entorhinal Principal Neurons Mediate Brain-stimulation Treatments for Epilepsy

**DOI:** 10.1016/j.ebiom.2016.11.027

**Published:** 2016-11-23

**Authors:** Zhenghao Xu, Yi Wang, Bin Chen, Cenglin Xu, Xiaohua Wu, Ying Wang, Shihong Zhang, Weiwei Hu, Shuang Wang, Yi Guo, Xiangnan Zhang, Jianhong Luo, Shumin Duan, Zhong Chen

**Affiliations:** a Department of Pharmacology, Key Laboratory of Medical Neurobiology of The Ministry of Health of China, College of Pharmaceutical Sciences, Zhejiang University, Hangzhou, Zhejiang, China; b Zhejiang Chinese Medical University, Hangzhou, Zhejiang, China; c Epilepsy Center, Second Affiliated Hospital, School of Medicine, Zhejiang University, Hangzhou, China

**Keywords:** AAV, adeno-associated virus, ADD, afterdischarge duration, ADT, afterdischarge threshold, ANOVA, analysis of variance, AP, antero-posterior, CaMKIIα, Calcium/calmodulin-dependent protein kinase II alpha, ChR2, Channelrhodopsin 2, EC, entorhinal cortex, eYFP, enhanced Yellow Fluorescent Protein, GST, generalized seizure threshold, HFES, High-frequency electrical stimulation, IN, interneuron, LFES, Low-frequency electrical stimulation, LMOL, lacunosum-moleculare layer, ML, mediolateral, PN, principal neuron, SNR, substantia nigra pars reticulate, TLE, Temporal lobe epilepsy, V, ventral, VGAT, vesicular GABA transporter, WT, wild-type, Epilepsy, Neuronal circuit, Optogenetics, Entorhinal cortex, Brain stimulation, Kindling

## Abstract

Brain stimulation is an alternative treatment for epilepsy. However, the neuronal circuits underlying its mechanisms remain obscure. We found that optogenetic activation (1 Hz) of entorhinal calcium/calmodulin-dependent protein kinase II α (*CaMKIIα*)-positive neurons, but not GABAergic neurons, retarded hippocampal epileptogenesis and reduced hippocampal seizure severity, similar to that of entorhinal low-frequency electrical stimulation (LFES). Optogenetic inhibition of entorhinal *CaMKIIα*-positive neurons blocked the antiepileptic effect of LFES. The channelrhodopsin-2-eYFP labeled entorhinal *CaMKIIα*-positive neurons primarily targeted the hippocampus, and the activation of these fibers reduced hippocampal seizure severity. By combining extracellular recording and pharmacological methods, we found that activating entorhinal *CaMKIIα*-positive neurons induced the GABA-mediated inhibition of hippocampal neurons. Optogenetic activation of focal hippocampal GABAergic neurons mimicked this neuronal modulatory effect and reduced hippocampal seizure severity, but the anti-epileptic effect is weaker than that of entorhinal LFES, which may be due to the limited spatial neuronal modulatory effect of focal photo-stimulation. Our results demonstrate a glutamatergic-GABAergic neuronal circuit for LFES treatment of epilepsy, which is mediated by entorhinal principal neurons.

## Highlights

• Activating (1 Hz) entorhinal *CaMKIIα*-positive neurons globally excite hippocampal GABAergic neurons and are antiepileptic.
• Inhibiting entorhinal *CaMKIIα*-positive neurons promote hippocampal seizures and blocks antiepileptic effects of LFES.
• Focally activating (1 Hz) hippocampal GABAergic neuron is antiepileptic, which is weaker than LFES.

Although the importance of the entorhinal-hippocampal circuit in TLE has been acknowledged for several decades, the role of entorhinal neurons in the hippocampal seizure remains obscure. By optogenetics and extracellular recording, Xu et al. discovered an entorhinal principal neurons mediated antiepileptic “glutamatergic-GABAergic” neuronal circuit for brain stimulation treatment of TLE. That is, electrical non-selectively or optical selectively activating (1 Hz) entorhinal *CaMKIIα*-positive neurons, a subtype of entorhinal principal neurons, globally excited hippocampal GABAergic neurons and had antiepileptic effects while optically inhibiting these neurons promoted hippocampal seizures and blocked the antiepileptic effect of entorhinal electrical stimulation treatment.

## 1 Introduction

Temporal lobe epilepsy (TLE) is one of the most common types of human epilepsy. The seizures associated with TLE typically arise in the hippocampus, and they are often resistant to antiepileptic drugs (Bialer and White, 2010, Schwartzkroin, 1994). Recurrent uncontrolled hippocampal seizures can result in learning and memory impairments (Lin et al., 2012) as well as sudden unexpected death in TLE patients (Espinosa et al., 2009). Although surgical resection of the epileptic focus may abolish hippocampal seizures, this approach is limited by the strict requirements for surgical selection, the risk of cognitive impairment (Bonelli et al., 2013), and the high recurrence rate for seizures after several years (Berg, 2011, Thom et al., 2010). Recently, brain stimulation has been proposed as an alternative option for treating epilepsy with advantages of reversibility and controllability (Fisher and Velasco, 2014). However, clinical translation of brain stimulation techniques requires an understanding of its underlying mechanisms, especially because electrical stimulation at specific brain areas can induce memory impairment (Coleshill et al., 2004), undesirable emotional responses (Lanteaume et al., 2007, Liu et al., 2012), and neuroendocrine disorders (Fink and Jamieson, 1974).

Low-frequency electrical stimulation (≤ 5 Hz, LFES) is a promising brain stimulation strategy for treating epileptic seizures. LFES that targeted the epileptic focus (Yamamoto et al., 2006) and the areas outside of the focus, such as the piriform cortex (Yang et al., 2006, Zhu-Ge et al., 2007), cerebellum (Wang et al., 2008), or white matter (Koubeissi et al., 2013), reduced seizure severity in TLE. Recently, increasing evidence has suggested that LFES has a short-term or even instantaneous antiepileptic effect during seizures; and it may therefore be possible that using “closed-loop” or seizure-triggered LFES (delivering LFES in response to seizure-related electroencephalographic activity) in a specific brain region could inhibit epileptic seizures while leaving other aspects of brain function less affected (Berenyi et al., 2012). Indeed, closed-loop optogenetic modulation of neuronal spiking in the epileptic focus was also shown to suppress seizures (Krook-Magnuson et al., 2013, Lopez-Meraz et al., 2004). Thus, increased attention is being paid to exploring epilepsy-related neuronal circuits to identify the optimal brain targets of brain stimulation (Krook-Magnuson and Soltesz, 2015, Paz et al., 2013).

The entorhinal cortex (EC) projects to nearly all parts of the hippocampus and may play important roles in both seizure initiation and seizure propagation in TLE (McIntyre and Gilby, 2008). By serving as a gateway, the EC may modulate the balance between inhibition and excitation in the hippocampus. Dysfunctions of the EC are frequently observed in epileptic brains, including atrophy (Bartolomei et al., 2005), hypometabolism (Goffin et al., 2009, Guo et al., 2009, Wang et al., 2014) and cell loss (Du et al., 1995). Each of these could potentially contribute to epileptogenesis and chronic seizures in the hippocampus. Our previous study showed that LFES at the EC had an antiepileptic effect when delivered during an epileptic afterdischarge duration (ADD) (Xu et al., 2010), suggesting that the EC may be a potential target for brain stimulation treatment for TLE. However, the neuronal circuitry underlying the mechanisms relevant to this process remains unclear. Therefore, in the present study, we focused on the classical but still mysterious entorhinal-hippocampal circuit. By using optogenetic techniques (Deisseroth, 2011), we demonstrate that an endogenic antiepileptic neuronal circuit, which is mediated by entorhinal *CaMKIIα*-positive neurons, is involved in the entorhinal LFES treatments for hippocampal seizures.

## 2 Materials and Methods

**Fig. 1:**
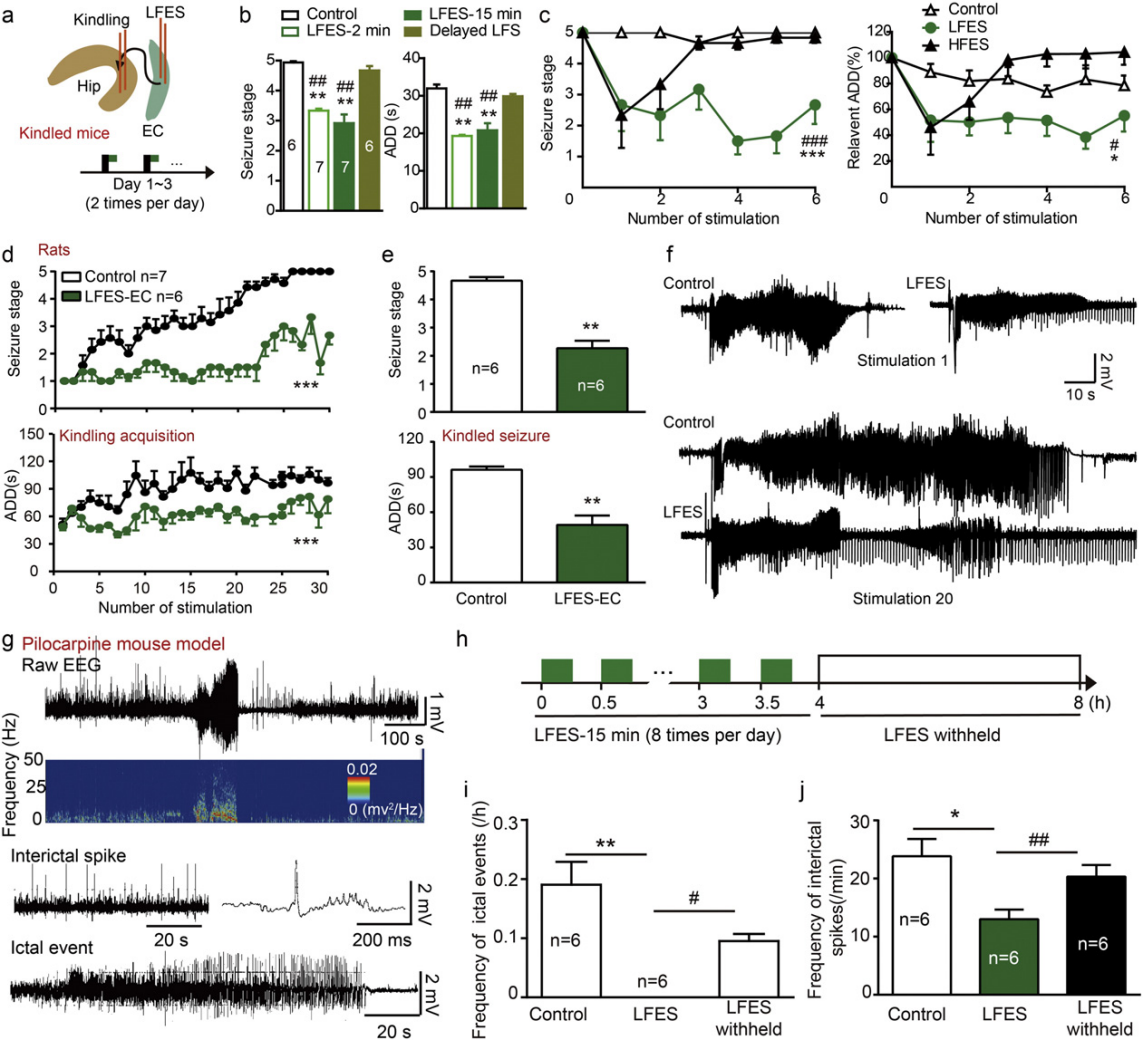
Entorhinal LFES reduced the severity of hippocampal seizures. a) Schematic diagram for kindling stimulation and LFES (low-frequency stimulation, 1 Hz, 2 or 15 min) delivery in kindled mice. Black rectangle indicates kindling stimulation while the green rectangle indicates LFES. b) The effect of entorhinal LFES on the seizure stage and ADD of hippocampal kindled seizures. LFES delivered 4 s after the kindling stimulation, while delayed LFES means LFES (1 Hz, 15 min) delivered after the termination of ADDs (about 30 s after the kindling stimulation). c) The effect of repeated entorhinal LFES (1 Hz, 0.3 mA) and high-frequency electrical stimulation (HFES, 50 Hz, 0.1 mA, 5 s on/5 s off) in kindled mice (two-way ANOVA for repeated measures followed by LSD *post hoc* test). d) Entorhinal LFES retarded the hippocampal kindling acquisition in rats (two-way ANOVA for repeated measures followed by LSD *post hoc* test). e) Entorhinal LFES reduces the severity of hippocampal kindled seizures in rats (one-way ANOVA for repeated measures followed by LSD *post hoc* test). f) Representative EEG showing entorhinal LFES shortened the ADD during kindling acquisition in rats. g) Representative EEG showing interictal and ictal events in a chronic established mouse pilocarpine model of epilepsy. Ictal events were defined as regular spike clusters with a duration of ≥ 20 s, spike frequency of ≥ 2 Hz and amplitude at least three times of the baseline EEG. Interictal spikes were defined as regular spikes with a spike frequency < 2 Hz and an amplitude at least three times of that of the baseline EEG. h) Schematic describing the delivery of entorhinal LFES. Green rectangle: LFES (1 Hz, 15 min); White rectangle: LFES withheld. i) Entorhinal LFES reduced the frequency of ictal events. (Wilcoxon signed rank test or Wilcoxon matched-pairs signed rank test for comparing to the LFES; Mann-Whitney *U* test was used for comparing LFES withheld to control). k) Entorhinal LFES reduced the frequency of interictal spikes (Student's *t*-test for comparing to the controls, and paired *t*-test for comparing to the LFES withheld). *p < 0.05, **p < 0.01 and ***p < 0.001 compared to the baseline or control. ^#^*p <* 0.05, ^##^*p <* 0.01 and ^###^*p <* 0.001 compared to HFES or LFES withheld.

**Fig. 2:**
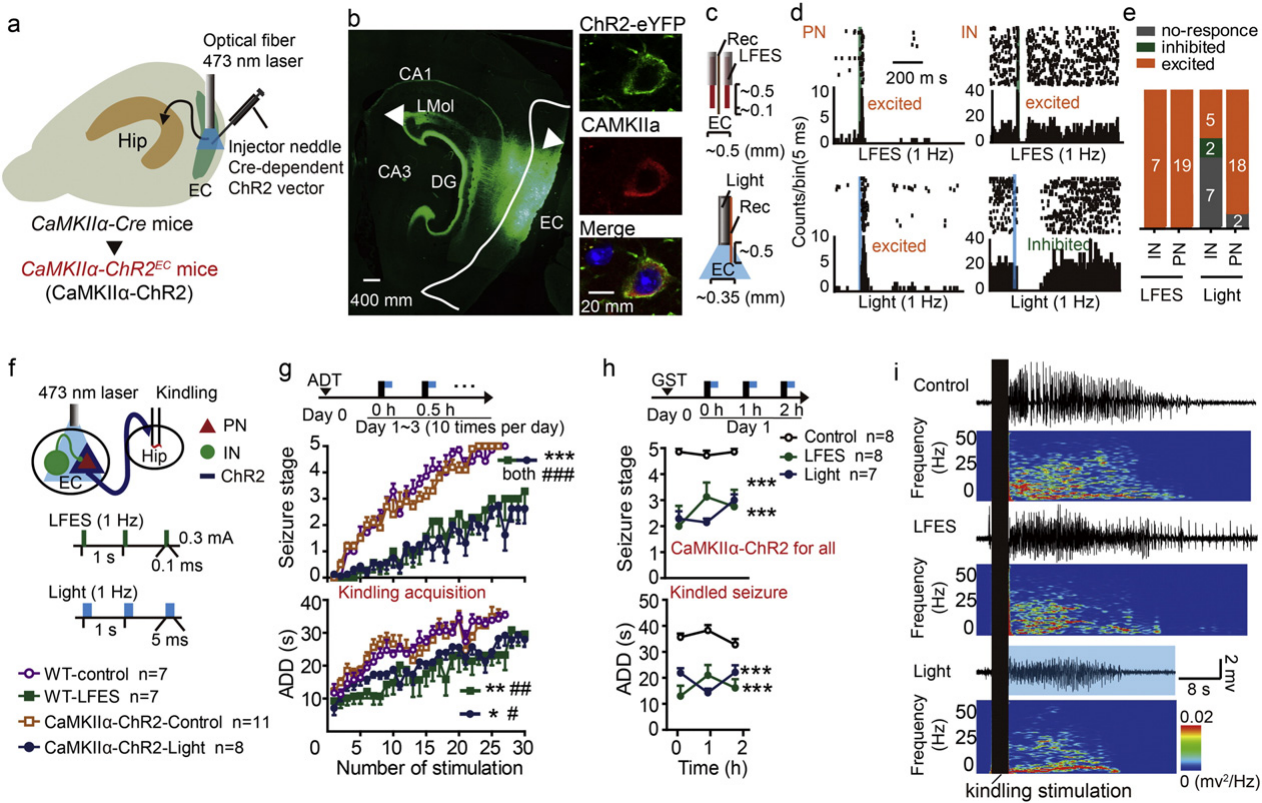
Activation of entorhinal CaMKIIα-positive neurons reduces the severity of hippocampal seizures. a) An AAV construct encoding Cre-dependent ChR2-eYFP (pAAV-EF1α-DIO-hChR2(H134R)-eYFP) was injected into the EC of *CaMKIIα-Cre* mice to generate *CaMKII*α*-ChR2*^*EC*^ mice (abbreviated to as “CaMKIIα-ChR2”). b) Histological images showing that ChR2.0-eYFP (triangles) was expressed in the EC and in the hippocampus (fibers). Immunohistochemical staining confirmed that ChR2-eYFP was expressed in CaMKIIα-positive neurons in the EC. c) The electrodes that would allow recording during electrical or optogenetic stimulation in the EC. d) Representative peri-event rasters showed the response of neurons to the LFES or photo-stimulation (1 Hz). Top Left: a PN excited by LFES; Top right: an IN exited by LFES; Bottom left: a PN exited by blue light; Bottom right: an IN inhibited by blue light. A neuron that showed a higher firing rate (at least three times higher than the baseline) immediately after stimulation was defined as an “excited” neuron, while a neuron that stopped firing (≥ 30 ms duration) within approximately 20 ms after stimulation was defined as an “inhibited” neuron. e) Population data showing that blue light (473 nm, 1 Hz) excited most PNs in the EC in 4 *CaMKII*α*-ChR2*^*EC*^ mice, and LFES excited both PNs and INs in 3 wild-type (WT) littermates. f**)** The schedule diagrams for the delivery of LFES and light (above) and the labels used for different groups (below). Black rectangle indicates kindling stimulation, the green rectangle indicates a pulse of LFES, the blue rectangle indicates a pulse of blue optical stimulation, and the yellow rectangle indicates a trail of yellow optical stimulation. Animals were grouped to match afterdischarge threshold (ADT), which was defined as the minimal intensity that produced an afterdischarge (≥ 5 s). g) Entorhinal light stimulation (473 nm, 1 Hz) and LFES retarded the development of behavioral stages of seizures and afterdischarge duration (ADD) during hippocampal kindling acquisition (two-way ANOVA for repeated measures followed by Least Significant Difference (LSD) *post hoc* test). Black rectangle indicates kindling stimulation while the blue rectangle indicates blue light stimulation. In kindling acquisition experiments, we used 10 stimulations a day for three days in normal mice. The CaMKIIα-ChR2 control received yellow light stimulation; and the WT-control received no photo-stimulation. h) Both entorhinal photo-stimulation and LFES lowered the seizure stage and shortened ADD in kindled mice (two-way ANOVA for repeated measures followed by LSD *post hoc* test). Black rectangle indicates kindling stimulation while the blue rectangle indicates blue light stimulation. Separate kindled mice were grouped to match generalized seizure threshold (GST), which was defined as the minimal current intensity required to elicit a generalized seizure. i) Representative EEGs. The black rectangle represents the kindling stimulation. Data are displayed as mean ± SEM. *p < 0.05, **p < 0.01 and ***p < 0.001 compared to the WT-control, and ^#^*p <* 0.05, ^##^*p <* 0.01 and ^###^*p <* 0.001 compared to the CaMKIIα-ChR2 control.

**Fig. 3:**
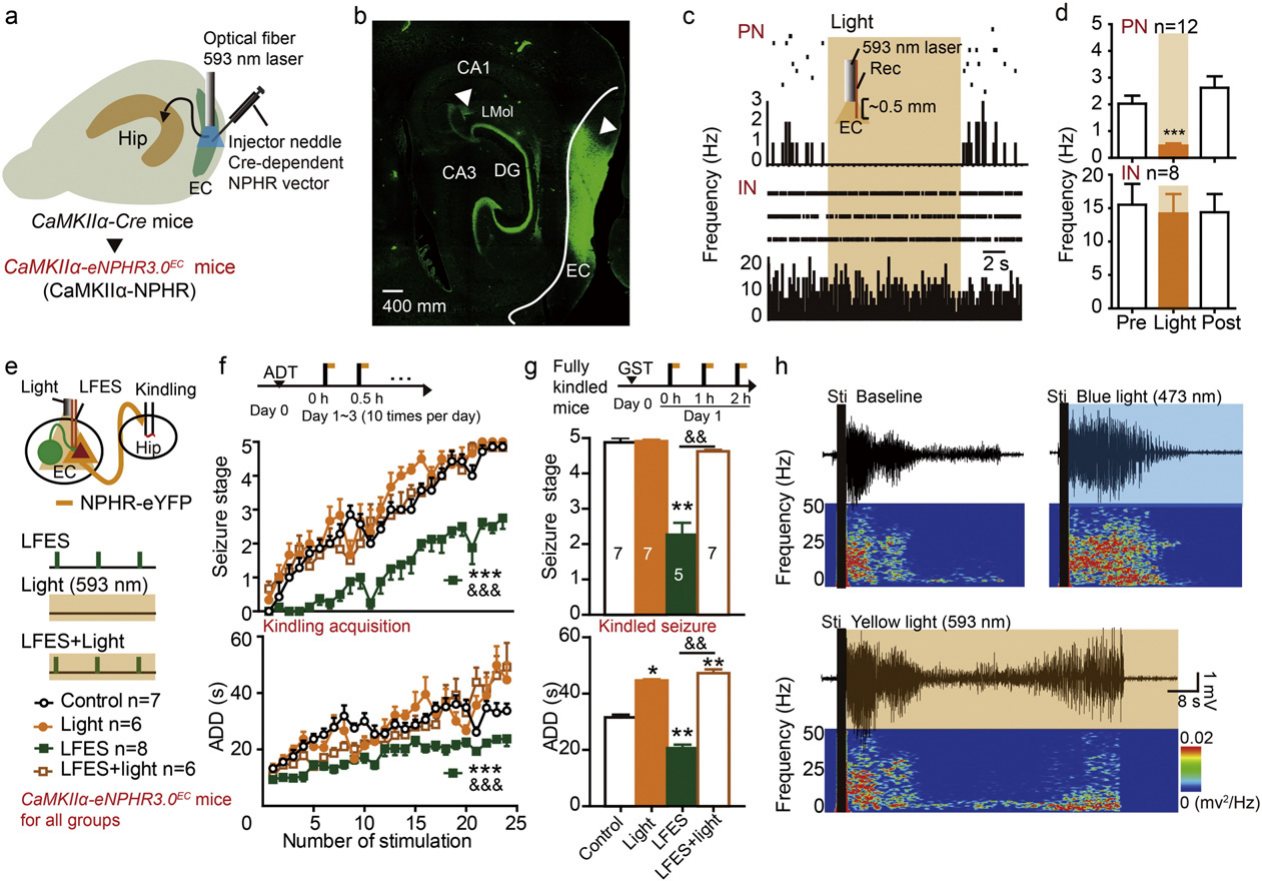
Inhibition of entorhinal PNs blocked the antiepileptic effect of entorhinal LFES. a) An AAV construct encoding Cre-dependent eNPHR3.0-eYFP (pAAV-EF1α-DIO-eNPHR3.0-eYFP) was injected into the EC of *CaMKIIα-Cre* mice to generate *CaMKIIα-NPHR*^*EC*^ mice (referred to as “CaMKII*α*-NPHR”). b) Histological images showing that eNPHR3.0-eYFP (triangles) was expressed in the EC and some hippocampus sub-fields (projection fibers). c) Representative peri-event rasters and d) statistical data form four mice showed that yellow light stimulation (593 nm, 20 Hz) reduced the firing rate of PNs but had no significant effect on INs in the EC (paired *t*-test). e) Schematic used in experiments to simultaneously deliver light and LFES to the EC during kindling. Black rectangle indicates kindling stimulation, the green rectangle indicates LFES and the yellow rectangle indicates yellow light stimulation. f) Entorhinal yellow light stimulation accelerated hippocampal kindling acquisition and attenuated the antiepileptic effect of entorhinal LFES in *CaMKIIα-NPHR*^*EC*^ mice (two-way ANOVA for repeated measures followed by LSD *post hoc* test). Black rectangle indicates kindling stimulation while the yellow rectangle indicates yellow light stimulation. The control and LFES groups received no photo-stimulation. g) Entorhinal yellow light stimulation promoted the kindled seizures and attenuated the antiepileptic effect of entorhinal LFES in *CaMKIIα-NPHR*^*EC*^ mice (Kruskal-Wallis followed by the Mann-Whitney *U* test). Black rectangle indicates kindling stimulation while the yellow rectangle indicates yellow light stimulation. The control and LFES groups received no photo-stimulation. h) Representative EEGs. Data are displayed as mean ± SEM. *p < 0.05, **p < 0.01 and ***p < 0.001 compared to the control or baseline; and ^&&^*p <* 0.01, ^&&&^*p <* 0.01 compared to the LFES group.

**Fig. 4:**
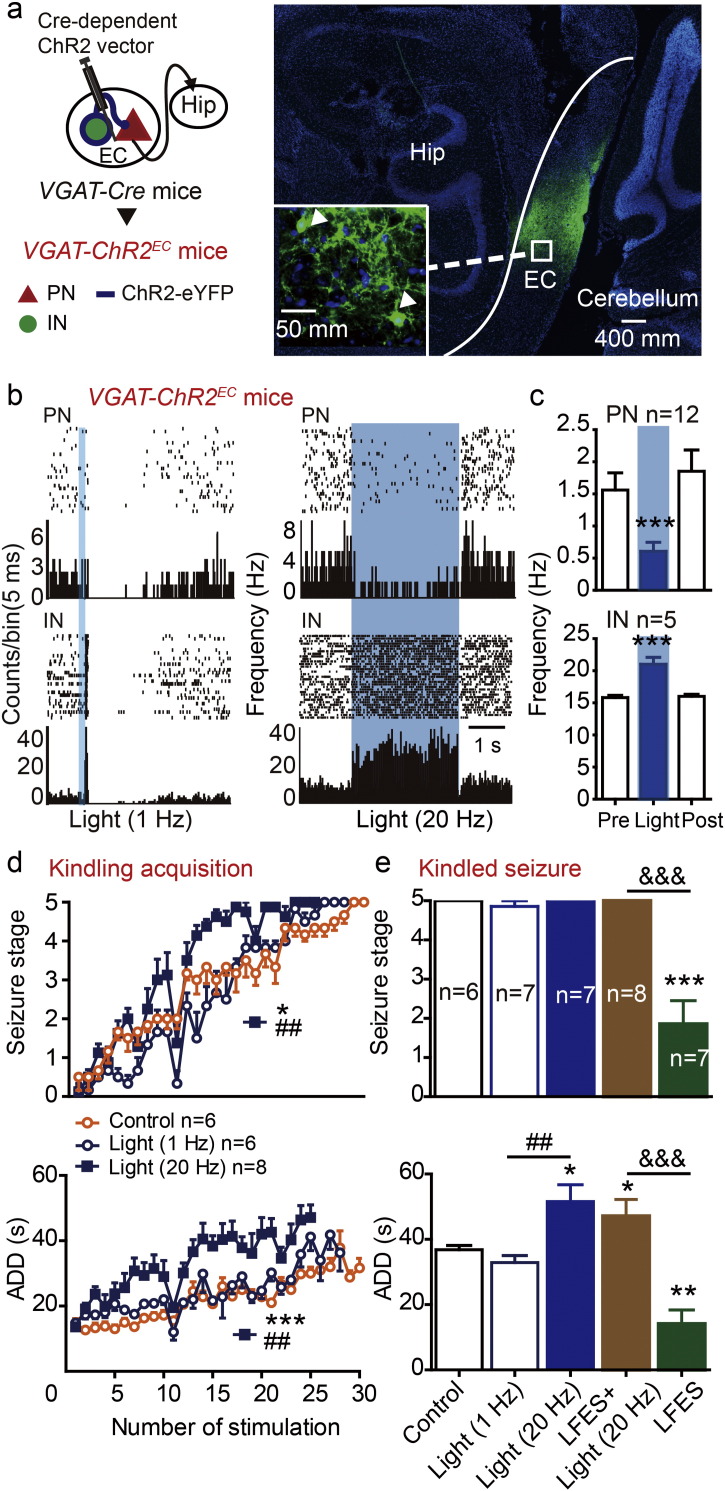
Activation of entorhinal GABAergic neurons promotes hippocampal seizures. a) Left: The generation of *VGAT-ChR2*^*EC*^ mice. Right: Histological data confirmed that ChR2-eYFP was expressed in the EC. b) Representative peri-event rasters blue light (473 nm, 1 Hz or 20 Hz, 5 ms per pulse) excited INs and inhibited PNs in the EC; c) statistical data for blue light (20 Hz) experiments (*n* = 3 mice). ***p < 0.001 compared to the baseline (paired *t*-test). d) Entorhinal blue light stimulation (20 Hz but not 1 Hz) promoted hippocampal kindling acquisition in *VGAT-ChR2*^*EC*^ mice. *p < 0.05, ***p < 0.001 compared to control, ^##^*p <* 0.01 compared to light (1 Hz) group (two-way ANOVA for repeated measures followed by LSD *post hoc* test). e) Entorhinal photo-stimulation (473 nm, 20 Hz) promoted seizure stage (above, Kruskal-Wallis followed by the Mann-Whitney *U* test) and the ADD (below, one-way ANOVA followed by LSD *post hoc* test) of kindled seizures and attenuated the effect of LFES in *VGAT-ChR2*^*EC*^ mice. *p < 0.05, **p < 0.01, ***p < 0.001 compared to control, ^##^*p <* 0.01 compared to light (1 Hz) group, ^&&&^*p <* 0.001 compared to the LFES group (). **p < 0.01, ***p < 0.001 compared to control.

**Fig. 5:**
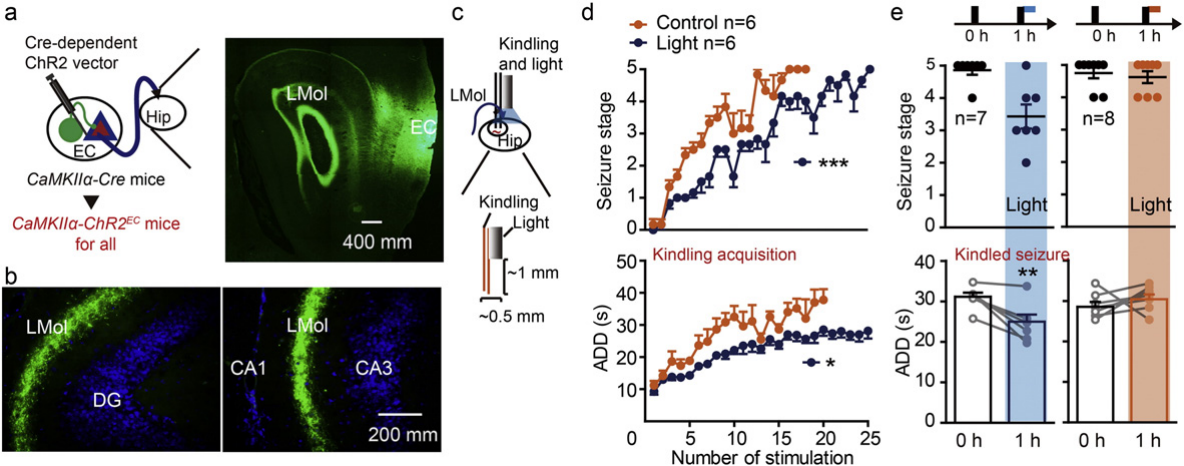
Activation of the projection fibers of entorhinal CaMKIIα-positive neurons reduces the severity of hippocampal seizures. a) ChR2-eYFP-labeled fibers mainly projected to the lacunosum-moleculare layer (LMOL) of the hippocampus in *CaMKIIα-ChR2*^*EC*^ mice. b) Representative ChR2-eYFP-labeled fibers in the section of hippocampus. c) The electrode design used for photo-stimulation of the hippocampal LMOL and kindling electrical stimulation of the ventral hippocampus. Electrical stimulation electrode for kindling is 1.0 mm lower than the optical fiber, which is just about the distance between the LMOL and the CA3 kindling focus. d) Low-frequency blue light stimulation (473 nm, 1 Hz) of the LMOL retarded the development of seizure stage (above) and ADD (below) during hippocampal kindling acquisition. (Two-way ANOVA for repeated measures followed by LSD *post hoc* test). The control group received yellow light stimulation (593 nm, 1 Hz). e) Low-frequency blue light stimulation (473 nm, 1 Hz) of the LMOL lowered the seizure stage (above) and shortened the ADD (below) in kindled mice (paired *t*-test). Black rectangle indicates kindling stimulation, the blue rectangle indicates blue light stimulation, and the yellow rectangle indicates yellow light stimulation. Mice were treated with blue light stimulation (473 nm, 1 Hz) and yellow light stimulation (593 nm, 1 Hz; yellow light self-control). Data are displayed as mean ± SEM. *p < 0.05, **p < 0.01 and ***p < 0.001 compared to the control or baseline.

**Fig. 6:**
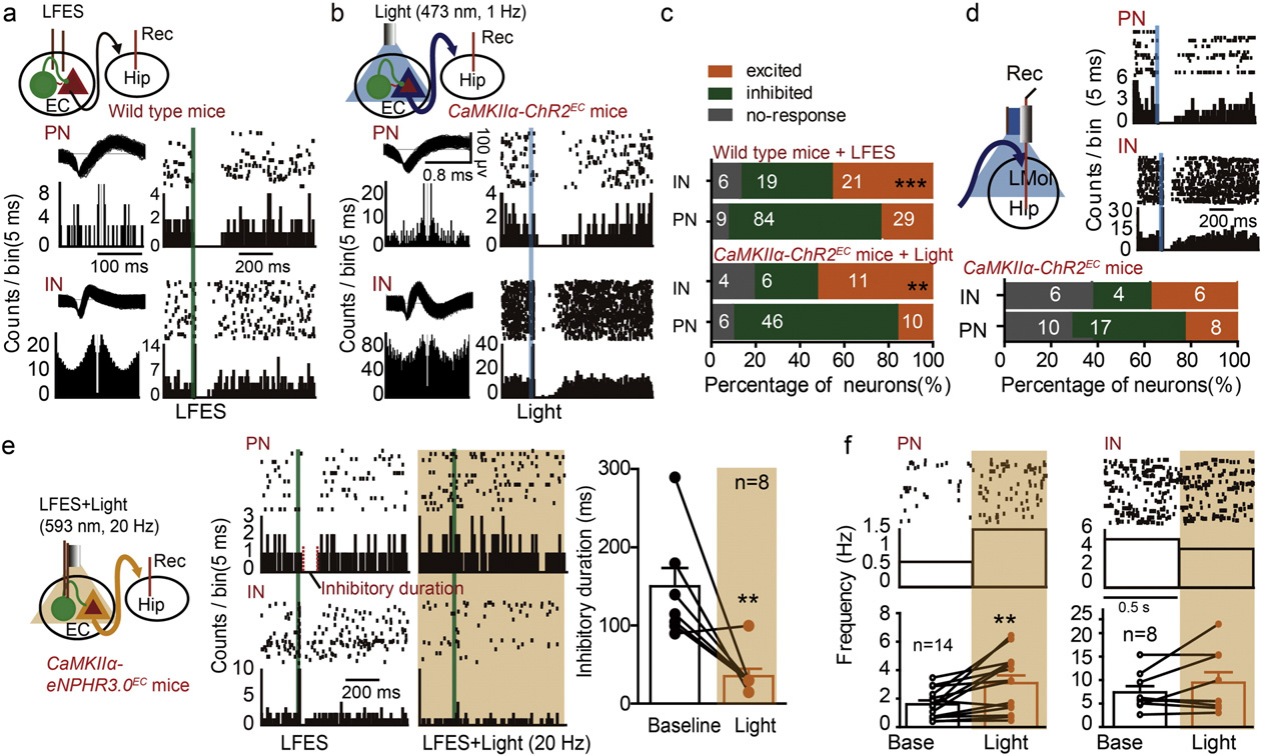
LFES primarily inhibits neurons in the ventral hippocampal CA3 by activating entorhinal CaMKIIα-positive neurons. a–b) Representative peri-event rasters showing the responses of PNs and INs in the ventral hippocampal CA3 area during LFES or photo-stimulation (473 nm, 1 Hz) in the EC; a: LFES in WT mice (*n* = 10); and b: photo-stimulation in *CaMKIIα-ChR2*^*EC*^ mice (*n* = 8). c) Distribution of hippocampal INs and PNs according to their response to entorhinal LFES or photo-stimulation (1 Hz) (*χ*^*2*^ test). d) Representative peri-event rasters and population data indicated that blue light stimulation at LMol modulated hippocampal neurons in *CaMKIIα-ChR2*^*EC*^ mice (*n* = 6). e) Entorhinal photo-stimulation (593 nm, 20 Hz) attenuated the inhibitory effect of entorhinal LFES in *CaMKIIα-eNPHR3*.*0*^*EC*^ mice (paired *t*-test). f) Entorhinal photo-stimulation (593 nm, 20 Hz) increased spiking frequency in hippocampal PNs (left) but not INs (right) in *CaMKIIα-eNPHR3*.*0*^*EC*^ mice (paired *t*-test). Data are displayed as mean ± SEM. **p < 0.01, ***p < 0.001 compared to PRI or baseline.

**Fig. 7:**
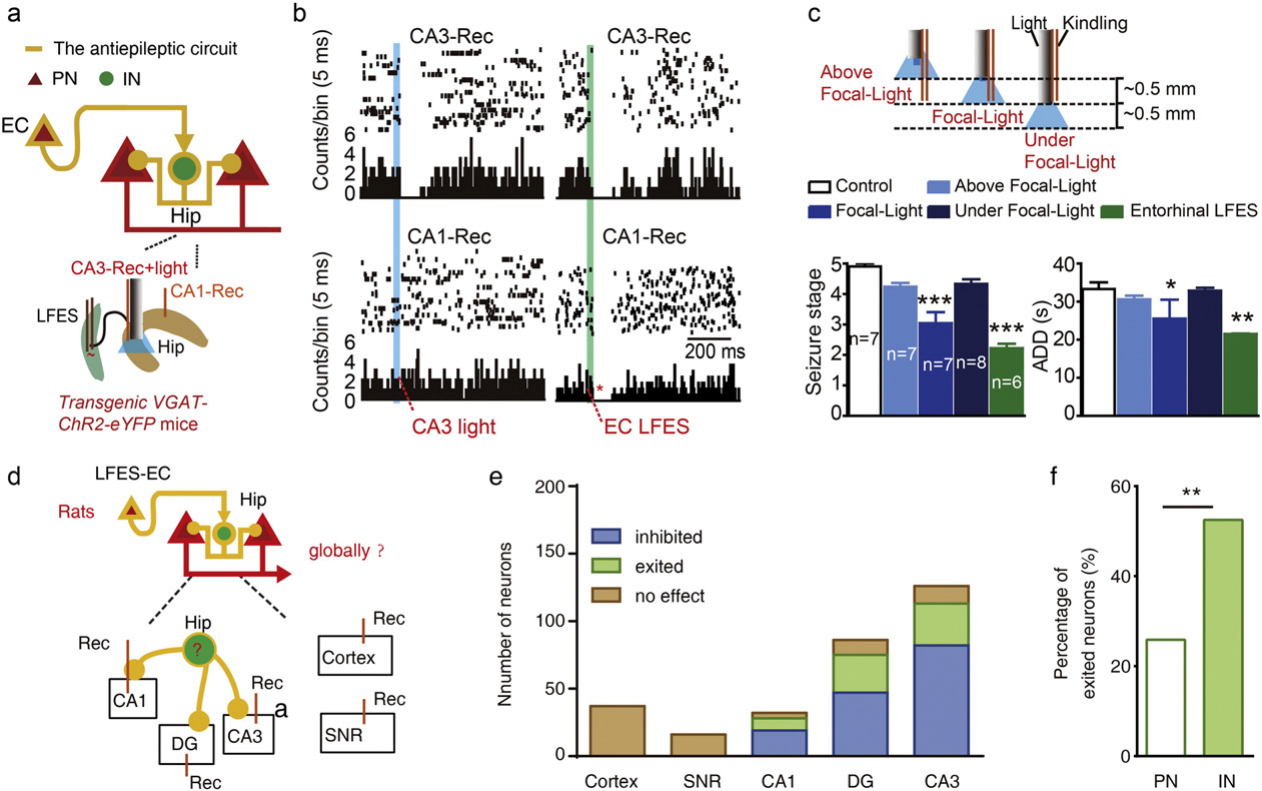
Activation of the hippocampal GABAergic system reduces the severity of hippocampal seizures. a) Left: Schematic of the antiepileptic circuit from the EC to the hippocampus (above), and the experiment strategy (below). b) Representative peri-event rasters showing that local low-frequency photo-stimulation at the kindling focus (CA3 subfield of hippocampus) modulated hippocampal CA3 (10 neurons) but not CA1 neurons (8 neurons), while entorhinal LFES modulated both (*n* = 3 transgenic VGAT-ChR2 mice). c) The antiepileptic effect of focal photo-stimulation was limited when the fiber slightly (0.5 mm) moved above or below the kindling focus, and entorhinal LFES showed stronger antiepileptic effect than focal photo-stimulation in kindled mice (Kruskal-Wallis followed by the Mann-Whitney *U* test was used for seizure stage; one-way ANOVA followed by LSD *post hoc* test was used for ADD). d) The strategy to recording neurons in rats. e) Entorhinal LFES modulated the neurons in all hippocampal subfields but not in the cortex above the hippocampus or the substantia nigra pars reticulate (SNR) (*n* = 10 rats). f) the proportion data showed that entorhinal LFES excited more INs than PNs in the hippocampus (*χ*^*2*^ test). Data are displayed as mean ± SEM. *p < 0.05, **p < 0.01 and ***p < 0.001 compared to control or PNs.

A general overview of the ultimate goals and its related experimental designs are listed as following:

A. Is LFES of the EC antiepileptic? – Test the effect of entorhinal LFES in hippocampal kindling and pilocarpine models of epilepsy (Fig. 1).
B. Is the antiepileptic effect of LFES mediated by entorhinal principal neurons (PNs) or their projection fibers to the hippocampus? – Optical activation or inactivation of entorhinal PNs through optogenetics (optical activation by ChR2 *vs* optical inactivation by eNPHR3.0; Fig. 2 for activation of entorhinal PNs, Fig. 3 for inactivation of entorhinal PNs and Fig. 5 for activation of their projection fibers).
C. Will optical excitation of entorhinal interneurons (INs) be proconvulsive? – Excitation of entorhinal INs through optogenetics (optical activation by ChR2; Fig. 4).
D. Which hippocampal neuronal types are predominantly affected by electrical or optical stimulation of entorhinal PNs? – Extracellular recordings of single neurons in the hippocampus during entorhinal LFES or optical activation of entorhinal PNs (Fig. 6).
E. Is the antiepileptic effect of LFES mediated by GABAergic neurons in the hippocampus? – Intrahippocampal injection of GABAergic receptor antagonists to block the anticonvulsive effects of entorhinal LFES and optical activation of hippocampal INs to inhibit seizures (Fig. 7).

### 2.1 Animals

Calcium/calmodulin-dependent protein kinase II alpha (*CaMKIIα*) Cre-recombinase mice (*CaMKIIα-Cre* mice; stock number 005359), vesicular GABA transporter (VGAT) Cre-recombinase mice (*VGAT-Cre* mice; 016962), VGAT-Channelrhodopsin 2 (ChR2)-enhanced Yellow Fluorescent Protein (eYFP) mice (transgenic *VGAT-ChR2-eYFP* mice; 014548) were genotyped according to the protocols provided by Jackson Laboratories. The use and care of the mice in accordance with the guidelines of the Animal Advisory Committee of Zhejiang University and the US National Institutes of Health Guidelines for the Care and Use of Laboratory Animals.

### 2.2 Stereotactic Viral Delivery and Surgery.

To selectively photo-modulate principal neurons or GABAergic neurons in the EC during *in vivo* behavioral experiments, Male mice (~ 2 months old) were microinjected with 0.3 μL of purified and concentrated adeno-associated virus (AAV, **~** 10^12^ infectious units per μL, Neuron Biotech Co.) into the right EC (antero-posterior (AP), − 4.6; mediolateral (ML), − 3.0; and ventral (V), − 4.2). AAV lists: pAAV2/8-EF1α-DIO-hChR2(H134R)-Eyfp and pAAV5-EF1α-DIO-engineered-halorhodopsin3.0 (eNPHR3.0)-eYFP. Negative littermates served as wild-type (WT) control. About 4 weeks after virus injection, the mice were fixed in a stereotaxic apparatus under anesthesia induced by sodium pentobarbital (60 mg/kg i.p., sigma), and bipolar electrodes or/and guide cannulas were implanted. Stereotactic viral delivery and surgery were based on the mouse brain atlas (Paxinos and Franklin, 2001). See the Supplement for details.

### 2.3 Hippocampal Kindling

After ~ 7 days of recovery, we determined hippocampal afterdischarge thresholds (ADT), the minimal intensity that produced an afterdischarge (AD, ≥ 5 s). AD was identified using a digital electroencephalograms (EEGs) system (NuAmps). Mice with an ADT > 200 μA were excluded. Then, mice received 10 kindling stimulations (400 μA, 20 Hz, 2 s) per day during 3–4 consecutive days. Behavioral seizures (seizure stage) were scored according to Racine's scale (Racine, 1972): (1) facial movement, (2) head-nodding, (3) unilateral forelimb clonus, (4) bilateral forelimb clonus and rearing, and (5) bilateral forelimb clonus and rearing and falling. Stages 4–5 were generalized seizures. In addition to seizure stage, ADD (AD duration) were recorded. When mice exhibited three consecutive stage 5 seizures, they were regarded as fully kindled. Fully kindled mice without treatment during kindling acquisition were used for kindled seizure experiments. These mice were confirmed to exhibit another three consecutive stage 5 seizures before experiments.

### 2.4 Electrical Entorhinal Stimulation

The protocol of electrical entorhinal stimulation was similar as described in a previous study (Xu et al., 2010). The electrical brain stimulation parameters were 100–300 μA, monophasic square-wave, 0.1 ms/pulse, 1–50 Hz. A schematic of the devise for electrical brain stimulation in the kindling model were showed in Fig. S1a. In kindling model entorhinal stimulation delivered 4 s after the kindling stimulation (about the appearance of AD), as shown in Fig. S1a and b. In kindling acquisition experiments, we used 10 stimulations a day for three days in normal mice. In kindled seizure experiments, we used two or three stimulations a day for one to three days as needed. In mouse pilocarpine model, LFES (1 Hz) was delivered using a cycle of 15 min ‘on’ and 15 min ‘off’ for 4 h every day, as shown in Fig. S1d.

### 2.5 Photo-stimulation

Laser light was delivered through a 200-μm diameter optic fiber connected to lasers (IKECOOL Laser) by rotary fiber-optic joints (Doric Lenses). The fiber was cut flat, and the laser power was adjusted to ~ 10.0 mW. About 15 min before experiment, the stylet was removed from the cannula, and the optic fiber was inserted and secured (~ 0.2 mm beyond). As shown in Fig. S1d, photo-stimulation was delivered about 4 s after the kindling stimulation and 2 min of duration as in LFES (Fig. S1b). Photo-activation used 473 nm, 1 or 20 Hz, 5 ms per pulse, while photo-inhibition used 593 nm, 20 Hz, 20 ms per pulse.

### 2.6 Extracellular Recording

Neuronal activity was sampled and analyzed as previously described (Kumar et al., 2013). Briefly, we recorded the neurons in the EC (AP, − 4.7; ML, − 2.75 to − 3.25; V, − 4– 4.5) and hippocampal CA3 (AP, − 2.9; ML, − 3; V, − 3.0) and CA1 (AP, − 1.5; ML, − 1.5; V, − 2) in mice,; and we also recorded the neurons in hippocampal CA3 (AP, − 5.5; ML, − 4.5; V, − 4.5– 5.5), DG (AP, − 3.5; ML, − 1.8; V, − 3.3– 3.8), CA1 (AP, − 3.5; ML, − 2; V, − 2.2– 2.7), cortex (AP, − 5.5; ML, − 4.5; V, − 0.8– 1.8) and SNR (AP, − 5.5; ML, − 2; V, − 6.5– 7) in rats. Recordings were made using a bundle of microelectrodes with 5–8 wires (25 μm, AM-Systems) with impedances of 1–2 MΩ as measured using an Omega-Tip-Z (World Precision Instruments, Inc.). Neuronal activity was sampled using a Cerebus acquisition system (Blackrock Microsystems; sampling rate 30 kHz, high-pass filtered at 250 Hz, low-pass filtered at 7500 Hz, and sorted online) grounded to screws above the cerebellum and referenced against a wire within the same brain area. To improve the signal-to-noise ratio, an online 50-Hz line-noise-cancelation algorithm was also applied. In general, we stably recorded unit spiking for at least 30 min with signal-to-noise ratio > 3:1. Recorded neuronal data were re-sorted offline using software (Plexon Inc.) to confirm the quality of the recordings, as described in previous studies (Brun et al., 2008, Schlesiger et al., 2015). Briefly, we carefully inspected the waveforms manually and removed the outlier events (an outlier was defined as a waveform that did not resemble any of the mean waveforms). We then applied several criteria before including a neuron in our data set: (i) a clean separation from all other units and background activity; (ii) a histogram for the inter spike interval distribution for the single neuron was not expected to show a refractory period, which was indicated by a lack of spikes occurring within one millisecond of each other.

Two independent criteria (spike width, and autocorrelation pattern) were applied to distinguish putative principal neurons (PNs) from interneurons (INTs), based on previous studies (Csicsvari et al., 1999, Le Van Quyen et al., 2008). Putative PNs were identified by their wide spike waveform (≥ 0.30 ms), and sharp autocorrelation; whereas putative INs were identified narrow spike waveform (≤ 0.30 ms), and flat autocorrelation. Non-typical INs (< 2 Hz) and PRI (> 10 Hz) were also excluded as unclassified neurons. Spike widths (peak to valley) were measured manually. Peri-event rasters, peri-event histograms, and autocorrelations were generated using Neuroexplorer 4.0 software (NEX Technologies Int. Inc). Putative axonal activity can be also excluded by its triphasic extracellularly recorded action potentials (Barry, 2015).

Because the classification of neurons by spike waveform and autocorrelation is empirical, we also accurately identified hippocampal INs using an optogenetic strategy in transgenic *VGAT-ChR2-eYFP* mice. The neurons entrained using 20-Hz blue light stimulation were identified as INTs, while neurons inhibited by 20-Hz blue light stimulation were identified as PNs.

The criteria used to define an “excited” or “inhibited” neuronal response to electrical or photo-stimulation were as follows: (a) a neuron that showed a higher firing rate (at least three times higher than the baseline) immediately after stimulation was defined as an “excited” neuron (or an “activation” response); (b) while a neuron that stopped firing (for at least 30 ms) within approximately 20 ms after stimulation was defined as an “inhibited” neuron (or an “inhibition” response).

### 2.7 Intrahippocampal Injection

A 30-gauge injection needle was inserted through the cannula and secured (~ 0.2 mm beyond). The injection duration for saline (1 μL), CGP35348 (5 μg/1 μL) and bicuculline (5 μg/1 μL) was 5 min and the needle was remained in the position for another 5 min before retraction. During extracellular recoding, drugs were slowly injected after neuronal spiking was stably recorded. For the kindling acquisition experiments performed in freely moving mice, the injection of the drugs was performed twice per day, approximately every 2.5 h (during 5 kindling stimulations). CGP35348 and bicuculline were purchased from Sigma-Aldrich.

### 2.8 Section Preparation and Imaging

Previously described methods were used for histological procedures (Han et al., 2014). Briefly, the mice were sacrificed and intracardially perfused with saline and 4% paraformaldehyde. Brain tissues were fixed in 4% paraformaldehyde overnight and then equilibrated in 30% sucrose. Using a freezing microtome (Leica), the tissues were cut into 30-μm sagittal sections. Some sections were processed for immunofluorescence with antibodies for GAD65/67 (1:500, Millipore AB1511) and CaMK2a (1:500, Abcam ab92332) and Alexa-594-conjugated secondary fluorescent antibodies (1 μg/mL, Jackson ImmunoResearch, 706-586-148). Sections were mounted on slides using Vectashield mounting medium (Vector Labs), and the fluorescence was assessed using an Olympus microscope (BX61).

### 2.9 Statistics

Analyses of group differences in kindling acquisition were performed using two-way analysis of variance (ANOVA) for repeated measures followed by the Least Significant Difference (LSD) *post hoc* test. *χ*^2^ test was used to compare the rate data. For seizure stage in kindled mice, we used Kruskal-Wallis test followed by Mann-Whitney *U* test to compare multiple groups, Mann-Whitney *U* test to compare the unpaired groups, and Wilcoxon matched-pairs signed rank test to compare the self-control data. Paired *t*-test was used to compare the self-control data when data with homogeneity of variance, otherwise Wilcoxon matched-pairs signed rank test. For other comparisons, Student's *t*-test or one-way ANOVA followed by the LSD *post hoc* test was used when data with homogeneity of variance, otherwise Kruskal-Wallis test or Mann-Whitney *U* test was used. In addition, Wilcoxon signed rank test was used instead of Student's *t*-test or Mann-Whitney *U* test when the data of one group is zero. All behavioral tests were conducted in a blinded manner. ADD measurements were not in a blinded manner because of the LFES related artificial events. The data are presented as the mean ± S.E.M. *p <* 0.05 was considered significant (two-tailed). The details of statistics for each figure please see Table S1

## 3 Results

### 3.1 Entorhinal LFES Reduced the Severity of Hippocampal Seizures

To investigate whether entorhinal LFES is antiepileptic, we tested the effect of entorhinal electrical stimulation on the severity of seizures in mice hippocampal kindling model (Fig. 1a). We found entorhinal LFES with 2 min of duration, which was delivered 4 s after the kindling stimulation, reduced the seizure stage and shortened the ADD of hippocampal kindled seizures in mice (*p* < 0.01 for both seizure stage and ADD compared to control, *n* = 7 for LFES with 2 min and *n* = 6 for control; Kruskal Wallis followed by Mann-Whitney *U* test was used; day Fig. 1b, left. Please see statistical detail in Table S1, similarly hereinafter). The antiepileptic effect of LFES of duration seemed to be dependent on the time of LFES delivery (LFES with 2 min of duration became ineffective when it was delivered just after the AD termination, the delayed LFES. *p >* 0.05 for both seizure stage and ADD compared to control, *n* = 6 for both delayed LFES and control; Kruskal Wallis followed by Mann-Whitney *U* test was used; Fig. 1b) but not dependent on the treatment duration (the effect of LFES with 2 min of duration is similar as that of LFES with 15 min of duration; *p >* 0.05 for both seizure stage and ADD, *n* = 7 for both group; Kruskal Wallis followed by Mann-Whitney *U* test was used; Fig. 1b). LFES suppressed hippocampal kindled seizures on all three days of stimulation (*p <* 0.001 for seizure stage and *p =* 0.029 for ADD compared to control, *n* = 6 for both LFES and control groups; two-way ANOVA followed by the LSD *post hoc* test was used; Fig. 1c), while HFS (20 or 50 Hz, 0.3 mA) in the EC can directly induce seizures (Fig. S3a), which is similar to the kindling phenomenon in the EC (Giacchino et al., 1984). Clinical use of HFES usually are sub-threshold (regarding seizure) HFES to avoid inducing seizures, so we also used sub-threshold HFES (50 Hz, 0.1 mA, 5 s on/5 s off) and found it slightly suppressed hippocampal kindled seizures in the short-term (*p >* 0.05 for both seizure stage and ADD compared to control, *n* = 6 for both HFES and control group; two-way ANOVA followed by the LSD *post hoc* test was used; Fig. 1c). Thus, those results at least indicated that LFES might be more effective than HFES for treatment of hippocampal seizures, and hence we further focused on entorhinal LFES.

To further confirm the antiepileptic effect of entorhinal LFES, we tested the antiepileptic effect of entorhinal LFES in rats. We found entorhinal LFES retarded the kindling acquisition (*p <* 0.001 for both seizure stage and ADD, *n* = 6 for LFES-EC group and *n* = 7 for control; two-way ANOVA followed by the LSD *post hoc* test was used; Fig. 1d) and reduced severity of hippocampal kindled seizures (*p* = 0.002 for seizure stage and *p* = 0.004 for ADD, *n* = 6 for both LFES-EC group and control; Mann-Whitney *U* test was used for seizure stage and Student's *t*-test was used for ADD; Fig. 1e) in rats. The representative EEGs shown in Fig. 1f demonstrate that entorhinal LFES shorten ADDs in rats. In addition, we also used another spontaneous chronic epileptic model, the mouse pilocarpine model of TLE. The representative EEGs of ictal and interictal evens in this chronic epileptic model are shown in Fig. 1g. Entorhinal LFES, delivered in a predefined schedule (Fig. 1h), reduced the frequency of ictal events (0.00 ± 0.00 for LFES *vs* 0.27 ± 0.054 for control, *p* = 0.031 by Wilcoxon signed rank test, *n* = 6 for each group; and 0.00 ± 0.00 for LFES *vs* 0.07 ± 0.01 for LFES withheld, *p* = 0.031 by Wilcoxon matched-pairs signed rank test, *n* = 6 for each group; Fig. 1i) and decreased the number of interictal spikes (13.00 ± 1.67 for LFES on *vs* 23.83 ± 2.97 for control, *p =* 0.016 by Student's *t*-test; and 13.00 ± 1.67 for LFES on *vs* 20.33 ± 1.99 for LFES withheld, *p =* 0.012 by paired *t*-test; *n* = 6 for each group; Fig. 1j). These preclinical data above confirm that EC is a promising effective target for LFES to treat hippocampal seizures in TLE.

In addition, to investigate whether entorhinal LFES affects seizure related behavioral deficits, we tested the effect of repeated entorhinal LFES on behavioral performance of three memory tests in kindled mice. Repeated entorhinal LFES relieved behavioral deficits in contextual fear memory (*p =* 0.010 compared to kindled-control, *n* = 11 for LFES EC group *vs n* = 11 for kindled-control; one-way ANOVA followed by the LSD *post hoc* test was used; Fig. S4a, left), cued fear memory (*p =* 0.037 compared to kindled-control, *n* = 11 for LFES EC group *vs n* = 11 for control; one-way ANOVA followed by the LSD *post hoc* test was used; Fig. S4a, right), passive avoidance tests (*p <* 0.001 compared to kindled-control, *n* = 11 for LFES EC group *vs n* = 11 for control; one-way ANOVA followed by the LSD *post hoc* test was used; Fig. S4b) and spatial object recognition (*p =* 0.014 compared to T0, *n* = 10 for LFES EC group; paired *t*-test was used; Fig. S4c), but it did not relieved behavioral deficits in novel object recognition tests (*p =* 0.986 compared to T0, *n* = 10 for LFES EC group; paired *t*-test was used; Fig. S4c). These preclinical data suggest EC might be a promising target for LFES relieving the cognitive deficits in TLE.

### 3.2 Activation of Entorhinal Principal Neurons Mimics the Antiepileptic Effect of LFES

To determine which sub-type of neurons in the EC contributed to the antiepileptic effect of entorhinal LFES, We transduced ChR2 into *CaMKIIα*-positive neurons, which are a subtype of PNs (principal neurons) (Yasuda and Mayford, 2006), by stereotactically injecting an adeno-associated virus (AAV) encoding a Cre-dependent ChR2 vector into the EC in *CaMKIIα-Cre* mice (referred to as *CaMKIIα-ChR2*^*EC*^ mice, Fig. 2a). Immunohistochemical images showed that ChR2.0-eYFP was expressed in *CaMKIIα*-positive neurons in the EC with their projection fibers in the hippocampus (Fig. 2b). We also used extracellular recordings of neural spiking in the EC to classify the recorded neurons according to their spike width and autocorrelation patterns (details see Fig. S2f), as described in previous studies (Csicsvari et al., 1999, Le Van Quyen et al., 2008). We designed electrodes that would allow recording during electrical or optogenetic stimulation in the EC (Fig. 2c) and measured the response of neuron to each pulse of electrical or optogenetic stimulation based on the peri-event histograms. Representative peri-event histograms of a PN excited by LFES, an IN excited by LFES, a PN excited by blue light and an IN inhibited by blue light were shown in Fig. 2d (each peri-event histogram indicated the same cell with repeated stimulations). We found that photo-stimulation (473 nm, 1 Hz) at the EC activated most putative entorhinal PNs (18 out of 20 from four *CaMKII*α*-ChR2*^*EC*^ mice, Fig. 2e) and a very small proportion of putative entorhinal INs (5 out of 19, which may be indirectly excited by the activated entorhinal PNs, Fig. 2e), and we additionally confirmed LFES (0.3 mA, 1 Hz) at the EC non-selectively activated both entorhinal INs (19 out of 19 from three WT mice, Fig. 2e) and PNs (7 out of 7, Fig. 2e).

To further test whether activation of entorhinal *CaMKIIα* positive neurons was sufficient to act anti-epileptic effect as LFES, we delivered low-frequency optical stimulation at the EC 4 s after the kindling stimulation as LFES in the hippocampal kindling mouse model (Fig. 2f). Interestingly, similar to the entorhinal LFES (*p <* 0.001 for seizure stage and *p =* 0.003 for ADD compared to WT control, *n* = 7 for both LFES and WT group; two-way ANOVA for repeated measures followed by LSD *post hoc* test was used; Fig. 2g), low-frequency photo-activation of entorhinal PNs retarded the development of seizure stage and shortened the ADD during kindling-induced epileptogenesis (kindling acquisition) (*p* < 0.001 for seizure stage and *p* < 0.01 for ADD compared to *CaMKIIα*-ChR2-Control, *n* = 8 for *CaMKIIα*-ChR2-Light group *vs n* = 11 for *CaMKIIα*-ChR2-Control; two-way ANOVA for repeated measures followed by LSD *post hoc* test was used; Fig. 2g). To confirm whether LFES suppresses kindling epileptogenic process or just suppressed the seizures, we measured the severity of hippocampal seizures on the fourth day (stimulation 31, when the LFES or light withheld) and compared it to the last stimulation on the third day (stimulation 30, when LFES or optical stimulation was on). We found the mean seizure stage (for LFES: 2.38 ± 0.50 *vs* 2.63 ± 0.57, *n* = 8; *p* = 0.750 by Wilcoxon matched-pairs signed rank test; for optical stimulation: 3.14 ± 0.60 *vs* 3.29 ± 0.78, *n* = 7; *p* > 0.999 by Wilcoxon matched-pairs signed rank test) and ADD (for LFES: 28.63 ± 1.34 *vs* 28.00 ± 2.26, *n* = 8, *p* = 0.804 by paired *t*-test, *t* = 0.258, df = 7; for optical stimulation: 27.43 ± 2.22 *vs* 29.57 ± 3.70, *n* = 7, *p* = 0.452 by paired *t*-test, *t* = 0.803, df = 6.) were similar between the two time points, indicating entorhinal LFES may suppress kindling epileptogenic process rather than just suppress the seizures**.**

In fully kindled mice, low-frequency photo-activation of entorhinal PNs also reduced the severity of kindled seizures (*p <* 0.001 for both seizure stage and ADDs, *n* = 7 for light group and *n* = 8 for control; two-way ANOVA for repeated measures followed by LSD *post hoc* test was used; Fig. 2h). To confirm whether LFES or light further suppresses kindled seizures after the LFES or light withheld, we measured the severity of hippocampal seizures 1 h after the LFES or light withheld. However, we found the seizure stage and ADD when LFES or optical stimulation was withheld are similar to the pre-LFES baseline (for seizure stage after LFES withheld compared to pre-LFES baseline: 4.86 ± 0.14 *vs* 5 ± 0, *n* = 7, *p* > 0.999 *by* Wilcoxon matched-pairs signed rank test; for ADD after LFES withheld compared to pre-LFES baseline: 32.71 ± 2.38 for withheld *vs* 30.86 ± 2.49, *n* = 7, *p* = 0.263 by paired *t*-test, *t* = 1.236, df = 6; for seizure stage after optical stimulation withheld compared to pre-stimulation baseline: 4.71 ± 0.18 *vs* 5.00 ± 0.00, *n* = 7, *p* = 0.500 *by* Wilcoxon matched-pairs signed rank test; for ADD after optical stimulation withheld compared to pre-stimulation baseline: 32.00 ± 1.96 *vs* 31.86 ± 1.88, *n* = 7, *p* = 0.957 by paired *t*-test, *t* = 0.057, df = 6) In addition, high-frequency photo-stimulation (473 nm, 20 Hz) of *CaMKIIα*-positive neurons in the EC can also directly induce seizures as HFES (Fig. S3b), while low-frequency photo-stimulation (473 nm, 1 Hz) did not induce any seizure-like events. The representative EEGs shown in Fig. 2i demonstrate that entorhinal LFES or blue light stimulation shorted ADDs in kindled mice. All of these results indicate that the activation of *CaMKIIα* positive neurons is sufficient to mimic the anti-epileptic effect of LFES in the EC.

### 3.3 Inhibition of Entorhinal Principal Neurons Promotes Hippocampal Seizures

To further test whether *CaMKIIα* positive neurons were required for the anti-epileptic effect of LFES, we stereotactically injected an AAV that encoded Cre-dependent engineered halorhodopsin 3.0 (eNPHR3.0) into the EC of *CaMKIIα-Cre* mice (referred to as *CaMKIIα-eNPHR3*.*0*^*EC*^ mice) to allow the selective photo-inhibition of entorhinal *CaMKIIα*-positive neurons (Fig. 3a). Histological staining showed that eNPHR3.0-eYFP was expressed in the EC (Fig. 3b). We also designed an electrode that would allow simultaneous electrical and optogenetic stimulation. The diameter of this electrode was < 0.5 mm, and both the stimulating (or recording) electrode and the optical fiber were implanted into the EC (Fig. 3c). Extracellular recordings confirmed that yellow light stimulation (593 nm, 20 Hz) selectively decreased the firing rate in putative entorhinal PNs in the *CaMKIIα-eNPHR3*.*0*^*EC*^ mice (2.03 ± 0.30 *vs* 0.45 ± 0.09, *p <* 0.001 compared to pre-stimulation baseline by paired *t*-test, *t* = 5.977, df = 11, *n* = 12 from four mice; Fig. 3c and d).

In the hippocampal kindling model, we tested the effect of photo-inhibiting entorhinal *CaMKIIα* neurons on kindling seizures and the antiepileptic effect of entorhinal LFES (Fig. 3e). We found that photo-inhibiting *CaMKIIα* neurons did not accelerated the acquisition of hippocampal kindling (*p =* 0.092 for seizure stage and *p =* 0.627 for ADD compared to control, *n* = 6 for light group *vs n* = 7 for control; two-way ANOVA for repeated measures followed by LSD *post hoc* test was used; Fig. 3f) but it prolonged ADD in hippocampal kindled seizures (*p =* 0.038 compared to control, *n* = 7 for both Light group and control; Kruskal-Wallis followed by the Mann-Whitney *U* test was used; Fig. 3g). Moreover, photo-inhibition of entorhinal *CaMKIIα* neurons blocked the antiepileptic effect of entorhinal LFES during kindling acquisition (*p <* 0.001 compared to the LFES group, *n* = 6 for LFES + light group *vs n* = 8 for LFES group; two-way ANOVA for repeated measures followed by LSD *post hoc* test was used; Fig. 3f) and in kindled mice (*p* = 0.003 compared to the LFES group, *n* = 7 for LFES + light group *vs n* = 5 for LFES group; Kruskal-Wallis followed by the Mann-Whitney *U* test was used; Fig. 3g). Representative EEGs that were recorded from the right hippocampus are shown in Fig. 3h. These results indicate that the activation of *CaMKIIα*-positive neurons is endogenously anti-epileptic, which is also required for the anti-epileptic effect of LFES.

### 3.4 Activation of Entorhinal GABAergic Neurons Promoted Seizures

GABAergic neurons are the other important sub-type of neurons in the EC. To investigate the role of entorhinal GABAergic neurons in hippocampal seizures and in the antiepileptic effect of entorhinal LFES, we stereotactically injected an AAV encoding Cre-dependent ChR2 into the EC of *VGAT-Cre* mice to selectively modulate GABAergic neurons as described in previous studies (Chow et al., 2010, Krook-Magnuson et al., 2014). We refer to these mice as *VGAT-ChR2*^*EC*^ mice (Fig. 3a, left). Histological data confirmed that ChR2-eYFPs were expressed in the EC (Fig. 3a, right). Extracellular recordings found that low-frequency blue light stimulation (473 nm, 1 Hz) excited entorhinal INs like LFES, but it inhibited the PNs which was different from LFES. High-frequency blue light stimulation (473 nm, 20 Hz) increased the firing rate of entorhinal GABAergic neurons (25.85 ± 3.91 for light *vs* 14.95 ± 3.04 for pre-stimulation baseline, *p <* 0.001 by paired *t*-test, *n* = 12 from three *VGAT-ChR2*^*EC*^ mice; Fig. 3b) and decreased the firing rate in entorhinal PNs (0.29 ± 0.34 for light *vs* 1.90 ± 0.16 for pre-stimulation baseline, *p <* 0.001 by paired *t*-test, *n* = 5 from three *VGAT-ChR2*^*EC*^ mice; Fig. 3b) in *VGAT-ChR2*^*EC*^ mice (see statistical details in Table S1).

In the hippocampal kindling model, high-frequency photo-activation of entorhinal GABAergic neurons (473 nm, 20 Hz) accelerated kindling acquisition (*p* = 0.011 for seizure stage and *p <* 0.001 for ADD compared to control, *n* = 8 for light group *vs n* = 6 for control; two-way ANOVA for repeated measures followed by LSD *post hoc* test was used; Fig. 4d) and prolonged the ADD of kindled seizures in *VGAT-ChR2*^*EC*^ mice (*p =* 0.049 compared to control, *n* = 6 for light group *vs n* = 6 for control; one-way ANOVA followed by LSD *post hoc* test was used; Fig. 4e), but low-frequency photo-activation (473 nm, 1 Hz) had no significant effect (Fig. 4d and e). Interestingly, photo-activation (20 Hz) attenuated the antiepileptic effect of entorhinal LFES in kindled mice (*p <* 0.001 for seizure stage and ADD compared to the LFES group, *n* = 8 for LFS + light group *vs n* = 7 for LFES group; Kruskal-Wallis followed by the Mann-Whitney *U* test was used for seizure stage and one-way ANOVA followed by LSD *post hoc* test was used for ADD; Fig. 4e), which may have been due to photo-activated (20 Hz) entorhinal GABAergic neurons inhibiting entorhinal PNs (Fig. 4b and c). In addition, in our preliminary experiment, we also found similar seizure promoting phenomenon of photo-activating entorhinal GABAergic neurons in transgenic *VGAT-ChR2-eYFP* mice (Fig. S5), in which ChR2 mainly expressed in GABAergic neurons globally and may be not as spatial specific as *VGAT-ChR2*^*EC*^ mice (Zhao et al., 2011) Thus, all of the above data indicated that activation of entorhinal PNs, but not GABAergic neurons, was required for the antiepileptic effect of entorhinal LFES.

### 3.5 Activation of the Fibers of Entorhinal Principal Neurons Is Antiepileptic

To investigate whether the hippocampal neuronal circuits are involved in antiepileptic mechanisms that were activated by entorhinal *CaMKIIα*-positive neurons, we applied low-frequency blue light stimulation (473 nm) to the lacunosum-moleculare layer (LMOL) of the hippocampus, which contained many ChR2-eYFP-labeled fibers that originated in entorhinal *CaMKIIα*-positive neurons, as shown by histology (Fig. 5a and b). We also designed an electrode that would allow optogenetic stimulation at the LMOL and electrical kindling stimulation at the hippocampal CA3 *in vivo* (Fig. 5c). Photo-stimulation (473 nm, 1 Hz) retarded kindling epileptogenesis (*p <* 0.001 for seizure stage and *p* = 0.013 for ADDs, *n* = 6 for both light and control group; two-way ANOVA for repeated measures was used; Fig. 5d) and reduced the severity of kindled seizures (seizure stage: 3.43 ± 0.37 for light *vs* 4.86 ± 0.14 for 0 h baseline, *p* = 0.063 by Wilcoxon matched-pairs signed rank test; ADD: 25.00 ± 1.75 for light *vs* 31.14 ± 1.01 for 0 h baseline, *p <* 0.004 by paired *t*-test; *n* = 7; Fig. 5e) in *CaMKIIα-ChR2*^*EC*^ mice. These data indicate that low-frequency activation of the hippocampal projection fibers of entorhinal *CaMKIIα*-positive neurons is sufficient to reduce the severity of hippocampal seizures. These results led us to next investigate whether entorhinal LFES modulated hippocampal focal neurons *via* the activation of entorhinal *CaMKIIα*-positive neurons.

### 3.6 LFES Inhibits Hippocampal Focal Neurons *via* Entorhinal PNs

To further explore the anti-epileptic micro-neuronal circuits in the hippocampus that are modulated by entorhinal *CaMKIIα*-positive neurons, we extracellularly recorded neural spiking in the ventral hippocampus and measured their response to each pulse of entorhinal stimulation (the kindling focus in our experiments and a common focus in TLE patients). By classifying the recorded neurons into putative PNs or INs, we found that both entorhinal LFES and the selective activation of entorhinal *CaMKIIα* neurons excited ~ 50% of the INs (21 out of 46 for LFES from ten WT mice and 11 out of 21 for photo-stimulation from eight *CaMKIIα-ChR2*^*EC*^ mice) and ~ 25% of the PNs (29 out of 122 for LFES and 10 out of 62 for photo-stimulation; Fig. 6a–c); and they also inhibited ~ 40% of the INs (19 out of 46 for LFES and 6 out of 21 for photo-stimulation) and ~ 70% of the PNs (84 out of 122 for LFES and 46 out of 62 for photo-stimulation; Fig. 6a–c). The proportion of excited INs were much more than PNs that induce by LFES or photo-stimulation (*p* < 0.001 and χ^2^ = 14.320 for LFES; and *p* < 0.01 and χ^2^ = 8.505 for photo-stimulation; Fig. 6c). We measured the duration of hippocampal PNs that inhibited by each pulse of entorhinal LFES (referred to inhibitory duration), and we found the inhibitory duration was intensity-dependent and seemed to be correlated with the strength of the antiepileptic effect induced by entorhinal LFES (Fig. S6). We also used transgenic *VGAT-ChR2-eYFP* mice to strictly identity the hippocampal GABAergic neurons by their response to the photo-stimulation, and confirmed similar results (Fig. S7). In addition, extracellular recordings showed that photo-stimulation (473 nm, 1 Hz) activating the projection fibers of entorhinal *CaMKIIα* neurons at LMOL modulated hippocampal neurons and that it seemed mainly to inhibit rather than excite them (*p* = 0.277 and χ^2^ = 1.182; Fig. 6d). Entorhinal LFES also had similar inhibitory effect on 13 out 25 hippocampal PNs in three mice of pilocarpine model (Fig. S8). These results indicate that the activation of entorhinal PNs excited a high proportion of hippocampal GABAergic neurons and primarily inhibited hippocampal PNs.

To investigate whether the effect of LFES is dependent on entorhinal PNs, we photo-inhibiting entorhinal *CaMKIIα* neurons during LFES. We found photo-inhibiting entorhinal *CaMKIIα* neurons (by eNPHR3.0) shortened the entorhinal LFES induced inhibitory duration in hippocampal PNs (35.00 ± 9.59 for light *vs* 150.00 ± 23.47 for pre-stimulation baseline, *p =* 0.005 compare to baseline by paired *t*-test, *t* = 4.004, df = 7, *n* = 8 from four *CaMKIIα-eNPHR3*.*0*^*EC*^ mice; Fig. 6e) and increased the firing rate of hippocampal PNs (3.60 ± 0.71 for light *vs* 1.75 ± 0.35 for pre-stimulation baseline, *p* = 0.002 compare to baseline by paired *t*-test, *t* = 3.849, df = 13, *n* = 14 from four *CaMKIIα-eNPHR3*.*0*^*EC*^ mice; Fig. 6f). Similarly, photo-activating entorhinal GABAergic neurons, which inhibit entorhinal *CaMKIIα* neurons, also shortened the entorhinal LFES induced inhibitory duration in hippocampal PNs (20.30 ± 3.65 for light *vs* 139.40 ± 10.72 for pre-stimulation baseline, *p <* 0.001 compare to baseline by paired *t*-test, *t* = 13.930, df = 8, *n* = 9 from two *CaMKIIα-eNPHR3*.*0*^*EC*^ mice; Fig. S9) and increased firing rate in hippocampal PNs (1.72 ± 0.23 for light *vs* 4.42 ± 0.80 for pre-stimulation baseline from two *CaMKIIα-eNPHR3*.*0*^*EC*^ mice, *p* = 0.003 compare to baseline by paired *t*-test, *t* = 4.122, df = 8, *n* = 9 from two *CaMKIIα-eNPHR3*.*0*^*EC*^ mice; Fig. S9). These results confirm that activation of entorhinal *CaMKIIα*-positive neurons, but not entorhinal GABAergic neurons, mediate the primarily inhibitory effect of LFES on hippocampal PNs.

### 3.7 Activation of Focal GABAergic Neurons Mimics the LFES

To further verify our hypothetical antiepileptic “glutamatergic-GABAergic” circuit for entorhinal LFES (Fig. 7a), we directly activated the GABAergic neurons in ventral hippocampal CA3 in transgenic *VGAT-ChR2-eYFP* mice. We found that photo-stimulation (473 nm, 1 Hz) at the kindling focus inhibited hippocampal PNs (Fig. 7b), retarded kindling epileptogenesis (*p* = 0.010 for seizure stage and *p* = 0.005 for ADDs compared to control-saline group, *n* = 6 for both light and control group; two-way ANOVA for repeated measures followed by LSD *post hoc* test was used; Fig. S10) and reduced the severity of kindled seizures (*p <* 0.001 for seizure stage and *p =* 0.017 for ADD, compare to control group, *n* = 7 for focal light *vs n* = 6 for control; Kruskal-Wallis followed by the Mann-Whitney *U* test was used for seizure stage and one-way ANOVA followed by LSD *post hoc* test was used for ADD; Fig. 7c) as LFES. Furthermore, similar to what was observed in LFES (Fig. S11), a focal injection of CGP35348 shortened the inhibitory duration of PNs that induced by focal low-frequency photo-stimulation (Fig. S10a), as well as attenuated antiepileptic effects of focal low-frequency photo-stimulation in transgenic *VGAT-ChR2-eYFP* mice (Fig. S10b). Low-frequency photo-stimulation seemed to have a weaker effect than the effect of entorhinal LFES (Fig. 7c). This may be due to the spatial limitations of focal photo-stimulation, which inhibited neurons in the kindling focus (CA3) but not in CA1 (Fig. 7b, left), whereas entorhinal LFES inhibited PNs neurons in both hippocampal CA1and CA3 areas (Fig. 7b, right). To further confirm that entorhinal LFES globally modulated hippocampal neurons, we recorded all subfields of the hippocampus as well as some brain areas outside of the hippocampus in rats during entorhinal LFES (Fig. 7d). Entorhinal LFES modulated the neurons in all hippocampal subfields but not in the cortex above the hippocampus or the substantia nigra pars reticulate in rats (Fig. 7e). The proportion data showed that entorhinal LFES excited more INs than PNs in the hippocampus (*p* < 0.01 and χ^2^ = 11.03; Fig. 7f). Supportively, the antiepileptic effect of low-frequency photo-stimulation (473 nm, 1 Hz) became much weaker when the fiber slightly (0.5 mm) moved above or below the kindling focus though it was still in the hippocampus (Seizure stage: for above focal-light: *p* = 0.062; for focal-light: *p* < 0.001; For under focal-light: *p =* 0.281; for LFES: *p =* 0.001. ADD: for above focal-light: *p =* 0.399; for focal-light: *p =* 0.017; for under focal-light: *p =* 0.882; for LFES: *p =* 0.001. All compared to control by Kruskal Wallis followed by Mann-Whitney U for seizure stage and one-way ANOVA followed by LSD *post hoc* test for ADD; Fig. 7c)

## 4 Discussion

**Fig. 8:**
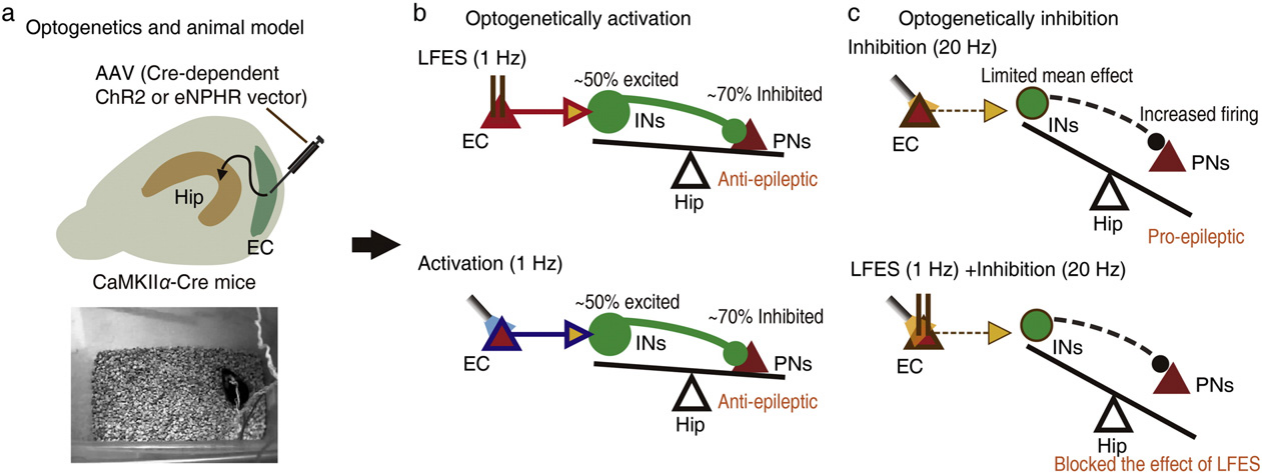
Hypothesized neuronal circuit mechanisms of entorhinal LFES treatment. a) The use of optogennetics in mice kindling model. b) Either optical selectively or electrical non-selectively activating (1 Hz) entorhinal CaMKIIα-positive neurons, a subtype of entorhinal PN, may tip the balance between hippocampal inhibition and excitation towards inhibitory or normal favor, because they globally excited ~ 50% hippocampal GABAergic neurons, inhibited ~ 70% hippocampal PN and reduced the severity of kindling seizures. c) In contrast, optical selectively inhibiting entorhinal PN may tip the balance between hippocampal inhibition and excitation towards excitatory favor, because it increase hippocampal the firing rate of hippocampal PN, promoted hippocampal seizures and blocked the antiepileptic effect of entorhinal LFES.

Although the importance of the entorhinal-hippocampal circuit in TLE has been acknowledged for several decades, the roles of the sub-types of entorhinal neurons in the hippocampal seizure process have remained obscure (Goldberg and Coulter, 2013). In the present study, we found that activation of entorhinal *CaMKIIα*-positive neurons, but not entorhinal INs, primarily inhibited hippocampal PNs and reduced the severity of hippocampal seizures (both epileptogenesis and kindled seizures, Fig. 8b). Activation of entorhinal GABAergic (20 Hz, but not 1 Hz) even promoted the severity of hippocampal seizures (both epileptogenesis and kindled seizures). Inhibition of entorhinal *CaMKIIα*-positive neurons primarily increased the activity of hippocampal PNs and promoted hippocampal seizures (Fig. 8c). These results provides directly *in vivo* data that entorhinal *CaMKIIα*-positive neurons have an endogenic antiepileptic effect on hippocampal seizures and that low frequency activation of these neurons may be a potential strategy for using brain stimulation to control hippocampal seizures.

The next question is how entorhinal *CaMKIIα*-positive neurons, a sub-type of excitatory PNs, exert their inhibitory and antiepileptic effects. To answer this question, we explored the downstream targets of entorhinal *CaMKIIα*-positive neurons. By using AAV transduced ChR2-eYFP into entorhinal *CaMKIIα*-positive neurons as described in previous studies (Deisseroth, 2011, Kohara et al., 2014), we found the LMol layer of the hippocampus contains many projection fibers of entorhinal *CaMKIIα*-positive neurons (Fig. 5); and selective activation of these fibers was sufficient to reduce the severity of hippocampal seizures, suggesting hippocampal neurons may be its downstream effectors. Extracellular recording also showed that activating entorhinal *CaMKIIα*-positive neurons excited a high proportion of putative INs (~ 50%) and inhibited about 70% of putative PNs in the hippocampus (Fig. 6), suggesting hippocampal INs may be the downstream inhibitory effectors of entorhinal *CaMKIIα*-positive neurons. This hypothesis was further supported by the following results: (a) intrahippocampal injection of GABAergic receptor antagonists (especially GABA_B_ receptor antagonist CGP35358) blocked both these inhibitory and antiepileptic effects of LFES (Fig. S11) which was mediated by entorhinal *CaMKIIα*-positive neurons; and (b) direct optogenetic activation of INs (1 Hz) in the hippocampal focus was sufficient to mimic both inhibitory and the antiepileptic effects (Fig. 7 and Fig. S10). Thus, taken together, our results demonstrate that entorhinal *CaMKIIα*-positive neurons and their modulated hippocampal INs from an antiepileptic “glutamatergic-GABAergic” circuit (Figs. 7a and 8), which may be the underlying neuronal basis that explains how *CaMKIIα*-positive neurons control hippocampal seizures.

Brain stimulation is a promising method for treating epilepsy. However, its clinical translation has been impeded by the obscure nature of its underlying mechanisms and the scarce effective targets (Udupa and Chen, 2015). Our results collectively showed that entorhinal LFES have an antiepileptic effect *via* non-selectively activating entorhinal *CaMKIIα*-positive neurons or its projection fibers and consequently inducing a global GABAergic inhibition in the hippocampus. This mechanism can partly explain that why delayed LFES has no antiepileptic effect and why the strength of antiepileptic effect of LFES with 2 min duration were similar to that with 15 min duration (Fig. 1b). Supportively, entorhinal LFES had a stronger antiepileptic effect in another pilocarpine induce spontaneous chronic epileptic mouse model in the LFES period than that in the LFES withheld period (Fig. 1i); and we did not find any long-term antiepileptic effect 1 h after 3 times of 2-min LFES treatments, when LFES or optical stimulation was withheld. Consistent with recent studies (Suthana et al., 2012), but differing from them in terms of stimulation frequency, repeated entorhinal LFES and low-frequency photo-activation of entorhinal PNs relieved the memory impairments in several behavioral tests in kindled mice (Fig. S3). Thus, the underlying entorhinal-hippocampal “glutamatergic-GABAergic” circuit may also be relevant for understanding the functions of the entorhinal cortex and the hippocampus in situations other than TLE. For example, lesioning the neurons in entorhinal layer III may results in hippocampal hyperactivity and impairs memory (Brun et al., 2008, Schlesiger et al., 2015). In addition, entorhinal LFES seemed to produce a stronger anti-epileptic effect than that of photo-stimulation of focal hippocampal GABAergic neurons, which may be due to the spatial limitations of focal photo-stimulation. Taken together, our results also confirmed that entorhinal LFES may be an efficacious option for brain stimulation treatment of temporal lobe epilepsy.

In addition, the traditional view of epileptic network activity mainly attributes it to excessive excitation and impaired inhibition in the brain, such as the loss of INs or the hyper-activation of surviving PNs. However, our results, as discussed above, present a different and more complex view of the epileptic network wherein excessive inhibition or impaired excitation of PNs in specific brain areas (such as in the EC) may also contribute to epileptic networks. Our results suggest a novel and important idea that excitation in specific brain areas can be inhibiting in another part of the circuit, and *vice versa*. This idea is different from previous theories that propose a phenomenon of “rebound excitation” to explain how “excessive inhibition” contributes to epilepsy in difference disease models, such as febrile status epilepticus (Chen et al., 2001) and other epilepsy models (Klaassen et al., 2006). In addition, our view of epileptic networks has several implications. First, it supports the view that the preferential loss of layer III PNs (Bartolomei et al., 2005, Du et al., 1995) and hypometabolism in the EC (Goffin et al., 2009, Guo et al., 2009, Wang et al., 2014) promote hippocampal epileptogenesis and establish seizures *in vivo*. Second, our proposal regarding epileptic networks may provide some clues for exploring why some excitatory drugs have antiepileptic effects in TLE models (Armstrong et al., 2009) while some inhibitory drugs induce rather than suppress seizures (Perucca et al., 1998, Schuele et al., 2005).

Overall, our results show that entorhinal *CaMKIIα*-positive neurons and the hippocampal INs form a “glutamatergic-GABAergic” antiepileptic circuit, which is involved in brain stimulation control of hippocampal seizures. Our work may facilitate the clinical translation of brain stimulation treatments for patients with epilepsy, and it may also add a new component to the current epileptic network theory.

## Acknowledgments and Funding Sources

This project was supported by grants from the National Natural Science Foundation of China (91332202, 81302749, 81221003, and 81471316) and the Program for Zhejiang Leading Team of S&T Innovation (2011R50014).

## Conflicts of Interest

None.

## Author Contributions

Z.H.X. and Z.C. designed experiments and co-wrote the manuscript. Z.H.X. and Y.W. performed most of the experiment, acquired most of the data presented, and performed the statistical analyses. B.C., W.W.H., and Z.X.N. performed in imaging. C.L.X., and Y.W. participated in the animal surgeries and carried out animal care. X.H.W. and Y.W. conducted the learning & memory studies. S.H.Z., S.W., and Y.G. assisted with statistical analyses and *in vivo* electrophysiological recordings. J.H.L., and S.M.D. assisted with optogenetics and contributed experimental suggestions.

## Appendix A Supplementary data

Supplementary material Image 1
