# Supplementary material for: Entorhinal Principal Neurons Mediate Brain-stimulation Treatments for Epilepsy
Source: eBioMedicine. 2016 Nov 23;14:148–60. doi: 10.1016/j.ebiom.2016.11.027 (PMC5161446; doi:10.1016/j.ebiom.2016.11.027)
Supplement: Supplementary file 1 — Supplementary material [file mmc1.pdf]

Supplementary data

Title: Entorhinal principal neurons mediate brain-stimulation treatment for epilepsy

1. Supplementary Materials and Methods

1.1 Stereotactic surgery

The mice were anesthetized using sodium pentobarbital (60 mg/kg, intraperitoneally (i.p.), Sigma-Aldrich). The stimulating electrode was made of twisted Teflon-coated tungsten wires (795500; diameter, 0.05 mm; A.M. Systems), and the guide cannulas had a 0.48-mm outer diameter and 0.34-mm inside diameter (62003, RWD Life Science). The animals were kept on a heating pad until recovery from anesthesia. Stereotactic implantations were based on the following coordinates in mm relative to bregma:

(a) To photo-activate entorhinal neurons in *VGAT-ChR2-eYFP* and *CaMKIIα-ChR2*^{EC} mice, we implanted a stimulating electrode into the right ventral hippocampus (AP, -2.9; ML, -2.75 to -3.25; ventral V, -3.0) based on the mouse brain atlas (Paxinos and Franklin, 2001), and a guide cannula into the right EC (AP, -4.36 to -4.84; ML, -2.76 to -3.24; V, -3.5) to aid in inserting an optical fiber (diameter, 200 μm; Thor Labs) during experiments (detailed below). The coordinates used for the insertion of the optical fiber tip were approximately AP, -4.6; ML, -3.0; and V, -3.7. This surgery protocol was also used for the kindling acquisition experiments in *VGAT-ChR2*^{EC} mice.

(b) For entorhinal electrical stimulation experiments, male wild-type littermates (WT, C57BL/6 background, 20-30 g) were used. A stimulating electrode was implanted into the right

ventral hippocampus for the kindling stimulation experiments, and another stimulating electrode was implanted into the right EC (AP, -4.6; L, -2.75 to -3.25; V, -4.2) for brain stimulation treatment experiments. For the rats, the coordinates used to reach the ventral hippocampus (AP, -4.8; L, -5.3; V, -6.3) and the EC (AP, -8.1; L, -4.0; V, -6.0) were based on the rat brain atlas (Paxinos and Watson, 2004).

(c) To apply simultaneous photo- and electrical stimulation to the EC in *CaMKIIα-eNPHR3.0*^{EC} and *VGAT-ChR2*^{EC} mice, a stimulating electrode was implanted into the right EC (AP, -4.7; ML, -2.75 to -3.25; V, -4.2) with a guide cannula (AP, -4.12 to 4.6; ML, -2.76 to -3.24; V, -3.5). The guide cannula was glued to the stimulating electrode, the tip of which extended 0.7 mm beyond the end of the cannula. In addition, another stimulating electrode was implanted into the right ventral hippocampus (AP, -2.9; ML, -2.75 to -3.25, V, -3.0) for kindling.

(d) To photo-activate entorhinal projection fibers in the lacunosum-moleculare layer (LMOL) of the hippocampus, we implanted a stimulating electrode into the right ventral hippocampus (AP, -2.9; ML, -2.75 to -3.25; V, -3.0) with a guide cannula (about AP, -3.0; ML, -2.76 to -3.24; V, -1.8) above the hippocampal LMOL. The stimulating electrode was glued to a guide cannula, and the tip of the stimulating electrode extended 1.2 mm beyond the end of the cannula.

(e) To photo-activate GABAergic neurons in the kindling focus, we implanted a bipolar electrode into the right ventral hippocampus (AP, -2.9; ML, -2.75 to -3.25; V, -3.0) of *VGAT-ChR2-eYFP* mice with a guide cannula. The cannula was glued to the stimulating electrode,

and the tip of the stimulating electrode extended 0.7 mm beyond the end of the cannula. The coordinates of the inserted optical fiber tip placed it approximately 0.5 mm above the tip of the kindling electrode (AP, -3.2; ML, -3.0; V, -2.5) during the experiments. In addition, another stimulating electrode was implanted into the right EC (AP, -4.6; L, -2.75 to -3.25; V, -4.2) to deliver LFES.

(f) To deliver drugs to the hippocampus during entorhinal LFES, we implanted a stimulating electrode into the right ventral hippocampus (AP, -2.9; ML, -2.75 to -3.25; V, -3.0) with a guide cannula (about AP, -3.0; ML, -2.76 to -3.24; V, -2.2). The guide cannula was glued to the stimulating electrode, and the tip of the stimulating electrode extended 0.8 mm beyond the end of the cannula. Finally, the coordinates of the injection needle tip placed it 0.5 mm from the cannula (i.e., 0.3 mm above the stimulating electrode tip (AP, -3.2; ML, -3.0; V, -2.7) during drug delivery.

1.2 Hippocampus EEG recording and analysis

Raw electroencephalogram (EEG) signals were recorded with band-pass filters spanning 0.5 to 100 Hz and sampled at 1000 Hz with a digital amplifier (NuAmps, Neuroscan System, USA). Recorded EEGs were direct-current shifted, digitally band-pass filtered from 0.5 to 100 Hz by Scan 4.5. Finally, the EEG data were imported into Labchart 7.0 software, where the power spectrum was performed using fast Fourier transform with a Hanning window. Ictal events were defined as regular spike clusters with a duration of ≥ 20 s, spike frequency of ≥ 2 Hz and amplitude at least three times of the baseline EEG. Interictal spikes were defined as

regular spikes with a spike frequency < 2 Hz and an amplitude at least three times of that of the baseline EEG.

1.3 Pilocarpine model of epilepsy

Scopolamine methyl bromide (2 mg/kg, i.p.; Sigma-Aldrich) was injected 30 min before pilocarpine administration to block peripheral side-effects. Pilocarpine hydrochloride (260 mg/kg, i.p.; Sigma) was then injected, and the animals were continuously monitored for behavioral seizures. Diazepam (4 mg/kg, i.p.; Sigma-Aldrich) was used to terminate status epilepticus after 60 min of continuous seizures to standardize the duration of seizure activity. At one month after pilocarpine administration, electrodes were implanted into the right ventral hippocampus and EC as described above.

After 7 days of recovery, the surviving mice were divided into two groups: one group received entorhinal LFES, and the other group received sham LFES. LFES was delivered using a cycle of 15 min ‘on’ and 15 min ‘off’ for 4 h every day for 7 days (Fig. S1d).

1.4 Behavioral tasks

Behavioral tasks, including object recognition memory tests, passive avoidance test and fear-conditioning tests, were performed 24 h after the completion of 7 days of LFES treatment (once per day) in kindled mice.

The novel object recognition and spatial object memory tests were performed as previously described (Favrais et al., 2011; Wang et al., 2016). The novel object recognition

and spatial object memory tests were performed as previously described[65]. Briefly, each animal was trained in a session by exposure to two identical, non-toxic objects (glass or hard plastic items) that were placed in the rear left and right corners of a 30 × 30 × 45 cm square arena. After this session, the animal was returned to its home cage for a 30-min retention interval. For novel object recognition tests, each animal was lowered into a testing arena in which one familiar object had been replaced by a novel object. For spatial object memory tests, each animal was lowered into a testing arena in which one familiar object had been moved. Each session was video-recorded, and the animal was given 4 min to explore. Exploration was defined as either pointing its nose toward the object at a distance of < 2 cm and/or touching it with the nose, whereas turning around, climbing, and sitting on an object were not considered to be exploration. The discrimination ratio was calculated as the time spent with the novel object divided by the total time spent in exploring either object. The objects to be discriminated were green cubes, blue pyramids, and red cylinders that were 5.8 cm high. The objects were randomized and counterbalanced across animals and groups. Objects and arenas were thoroughly cleaned with 70% ethanol between trials to remove olfactory cues.

Passive avoidance testing was conducted using a 3-day paradigm, as previously described (King et al., 2003). Day 1: mice explored the apparatus for 5 min. Day 2 (training): mice were placed in the lighted chamber, and their latency to enter the dark chamber was recorded for a maximum of 5-min. On entering the dark chamber, the mice received a mild, inescapable foot-shock (0.5 mA, 2 s) and were then kept in the dark chamber for 1 min. Day 3 (test): the mice were placed in the lighted chamber, and their latency to enter the dark chamber was recorded.

Fear-conditioning procedures were performed as previously described (Liu et al., 2007). Briefly, mice were given two 3-min preconditioning handling sessions. For training on day 1, the mice were placed in the testing chamber, and a training tone (84 dB, 2.8 kHz, 30 s) was activated 2 min later. Two seconds before the end of the tone, a 2-s foot-shock (1 mA) was delivered. The mouse was returned to its home cage 30 s after the shock. For contextual fear testing, on day 2, the mice were placed back into the same chamber for 5 min, and the percentage of time spent freezing was estimated by scoring the presence or absence of non-respiratory movement every 5 s during 5 min. For cued fear testing on day 3, the mice were placed into a different box that contained visual, tactile, and olfactory cues that were distinct from those in the training apparatus. The training tone (84 dB, 2.8 kHz, 3 min) was activated 2 min later, and the percentage of time spent freezing was estimated during 3 min with the training tone.

2. Supplementary results

2.1 Entorhinal LFES relieved behavioral deficits in kindled mice (for Fig. S4)

To investigate whether entorhinal LFES affects seizure related behavioral deficits, we tested the effect of repeated entorhinal LFES on behavioral performance of three memory tests in kindled mice. We found behavioral performance on these memory tests is impaired in kindled mice, including contextual fear memory (p = 0.015 compared to sham-control, n = 11 for both kindled-control and sham-control groups; one-way ANOVA followed by the LSD *post hoc* test was used; Fig. S4a, left), cued fear memory (p = 0.013 compared to sham-control; n = 11 for both kindled-control and sham-control groups; one-way ANOVA followed by the LSD *post hoc* test was used; Fig. S4a, right), passive avoidance memory tests (p = 0.004 compared to sham-control; n = 10 for kindled-control and n = 11 for sham-control; one-way ANOVA followed by the LSD *post hoc* test was used; Fig. S4b), novel object recognition (p = 0.776 compared to baseline T0 for kindled group, while p = 0.033 compared to baseline T0 for sham group; n = 11 for kindled-control and n = 10 for sham-control group; paired t test was used; Fig. S4c) and spatial object recognition (p = 0.776 compared to baseline T0 for kindled group, while p = 0.003compared to baseline T0 for sham group, n = 11 for kindled-control and n = 10 for sham-control group; paired t test was used; Fig. S4c). Repeated entorhinal LFES relieved behavioral deficits in contextual fear memory (p = 0.010 compared to kindled-control n = 11 for LFES EC group *vs* n = 11 for kindled-control; one-way ANOVA followed by the LSD *post hoc* test was used; Fig. S4a, lef), cued fear memory (p = 0.037 compared to kindled-control; n = 11 for LFES EC group *vs* n = 11 for control; one-way ANOVA followed by the

LSD *post hoc* test was used; Fig. S4a, right), passive avoidance tests ($p < 0.001$ compared to kindled-control; n = 11 for LFES EC group *vs* n = 11 for control; one-way ANOVA followed by the LSD *post hoc* test was used; Fig. S4b) and spatial object recognition ($p = 0.014$ compared to T0 by paired t test; n = 10 for LFES EC group; Fig. S4c), but it did not relieved behavioral deficits in novel object recognition tests ($p = 0.906$ compared to T0 by paired t test; n = 10 for LFES EC group; Fig. S4c). It has been found that the novel object memory may be the perirhinal cortex dependent rather than hippocampal dependent (Kinnavane et al., 2016). LFES relieved behavioral deficits in fear memory, passive avoidance memory and spatial object recognition test but not in novel object recognition test, suggesting there are important limits to LFES treatment depending on the type of cognitive deficits.

Similar to LFES, repeated photo-activation (473 nm, 1 Hz) of entorhinal CaMKIIα neurons also relieved behavioral deficits in contextual fear memory ($p = 0.018$ compared to kindled-control, n = 5 for light-EC group and n = 11 for both kindled-control; one-way ANOVA followed by the LSD *post hoc* test was used; Fig. S4a, left), cued fear memory ($p = 0.015$ compared to kindled-control, n = 5 for light-EC group and n = 11 for both kindled-control; one-way ANOVA followed by the LSD *post hoc* test was used; Fig. S4a, right) and passive avoidance tests ($p < 0.001$ compared to kindled-control, n = 6 for light-EC group and n = 10 for both kindled-control; one-way ANOVA followed by the LSD *post hoc* test was used; Fig. S4c).

2.2 Activation of entorhinal GABAergic neurons promoted hippocampal seizures in transgenic VGAT-ChR2-eYFP mice (for Fig. S5)

GABAergic neurons are the other important sub-type of neurons in the EC. To investigate the role of entorhinal GABAergic neurons in hippocampal seizures and in the antiepileptic effect of entorhinal LFES, we first used transgenic *VGAT-ChR2-eYFP* mice[38]. As shown in Fig. S4, low-frequency blue light stimulation (473 nm, 1 Hz) in the EC had no antiepileptic effect in *VGAT-ChR2*^{EC} mice, although it did activate interneurons (INs) similar to LFES (Fig. S5b). High-frequency blue light stimulation (473 nm, 20 Hz) inhibited entorhinal principal neurons (PNs, Fig. S5c), accelerated hippocampal kindling acquisition (p = 0.026 for seizure stage compared to control, two-way ANOVA for repeated measures followed by LSD *post hoc* test was used. Fig. S5d) and prolonged the ADD of kindled seizures (48.88 ± 4.33 for light *vs* 29.25 ± 3.06 for pre-stimulation baseline, $p < 0.001$, paired t test, t = 5.921 df =7, n = 8; Fig. S5e). Representative EEGs showing that blue light stimulation prolonged the ADD of kindled seizures in *VGAT-ChR2-eYFP* mice (Fig. S5f).

Because ChR2-eYFP was globally expressed in the brain, especially in the cerebellum, photo-stimulation of the EC may activate the pass by fibers as well as the cerebellar cells/axons in transgenic *VGAT-ChR2-eYFP* mice. Hence, to exclude these potentially confounding factors, we stereotactically injected an AAV encoding Cre-dependent ChR2 into the EC of *VGAT-Cre* mice to selectively modulate GABAergic neurons, as described in Fig. 2.

2.3 LFS at the EC modulated optogenetic identified hippocampal neurons in VGAT-ChR2 mice (for Fig. *S7)*

As classification of neurons by the spike waveform and autocorrelograms seems empirical, we further identified hippocampal INs accurately using an optogenetic strategy in *VGAT-ChR2-eYFP* mice. The neurons that entrained by 20 Hz blue light stimulation were identified as INs (named light+ neuron, Fig. S7a), while neurons that inhibited by 20 Hz blue light stimulation were identified as non-INs (named light- neuron, Fig. S7a). Finally, 11 INs s and 36 non- INs were recorded. When delivering LFS at the EC, 7 INs showed excited response, 4 INs showed inhibited response, 9 non-INs showed excited response and 25 non- INs showed inhibited response (Fig. S7b). The ratio of type2/type1 in INs is much higher than that in non-INs (p = 0.029, χ^2 test, χ^2 = 5.601; Fig. S7c).

2.4 Hippocampal GABA receptor involved in the effects of LFES (for Fig. S11)

It has been reported that inhibition of focal PNs in the hippocampus inhibits seizures (Krook-Magnuson et al., 2013; Tonnesen et al., 2009), and selective activation of hippocampal PNs directly induces seizures (Alexander et al., 2009). These previous results, together with our extracellular recording data in Fig. 6, suggest that it is possible that entorhinal LFES induces its antiepileptic effect by activating specific inhibitory mechanism in the hippocampus (such as the hippocampal GABAergic system). To investigate whether the hippocampal GABAergic system contributes to the inhibitory and antiepileptic effects of entorhinal LFES, we injected GABA receptor antagonists into the hippocampus (Fig. S11a). We found that

injecting the GABA receptor antagonists bicuculline (5 μg in 1 μL, a critical sub-convulsive dose) or CGP35348 (5 μg in 1 μL, a sub-convulsive dose) shorted the inhibitory duration of hippocampal neurons which induced by entorhinal LFES (p = 0.038 for bicuculline compare to baseline, n = 13, paired t test, t = 2.438, df = 9. p < 0.001 for CGP35348 compare to baseline, n = 6, paired t test, t = 8.490, df = 5; Fig. S11c). CGP35348 also abolished the antiepileptic effect of entorhinal LFES (Fig. S11d). Interestingly, CGP35348 seemed to be much more effective than bicuculline in attenuating both the inhibitory and antiepileptic effects of entorhinal LFES (two-way ANOVA for repeated measures followed by LSD *post hoc* test; Fig. S11c and d). In addition, a high dose of CGP35348 (20 μg in 1 μL) reversed the antiepileptic entorhinal LFES into seizure-promoting. These results indicate that the hippocampal GABAergic system is required for the anti-epileptic effect of LFES.

2. Supplementary Figure

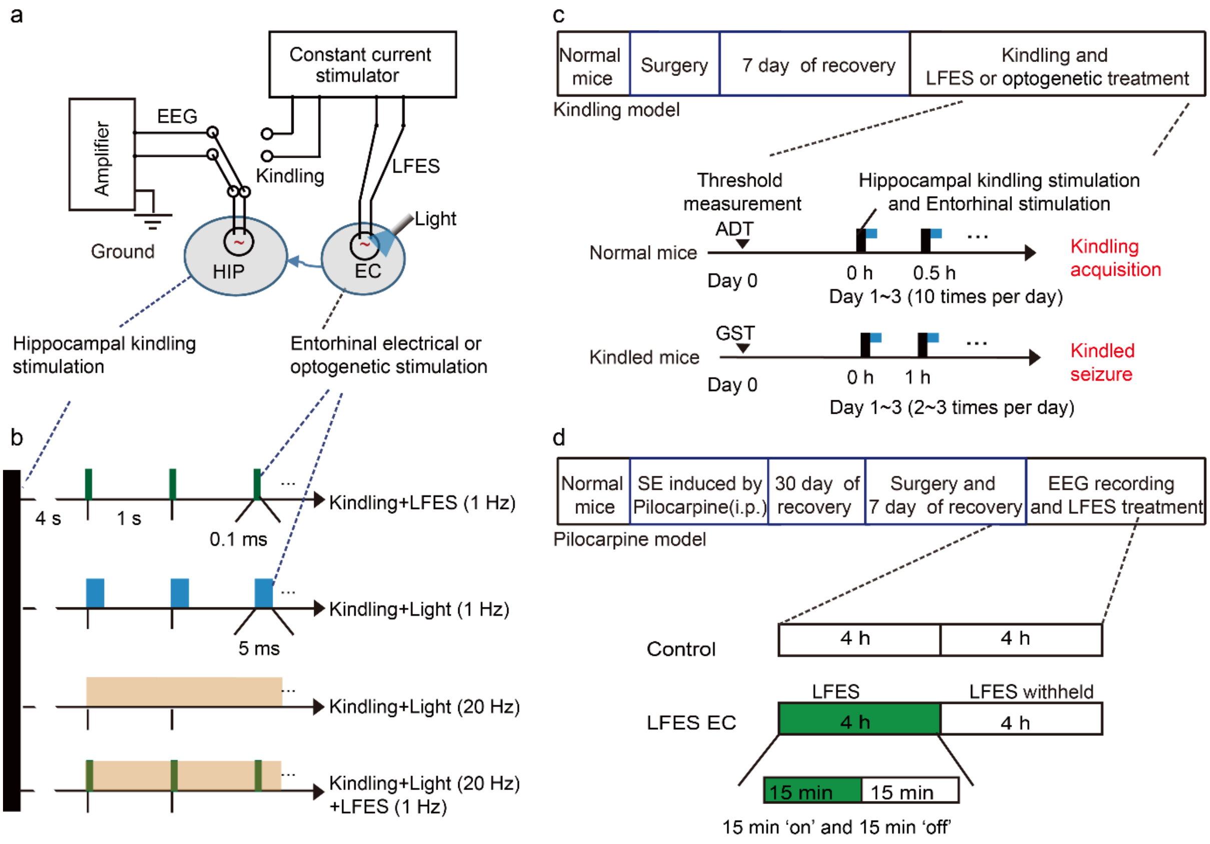

Fig. S1. Schematic of experimental protocol. a) A schematic of the devise for kindling and electrical brain stimulation. b) Examples of LFES, low-frequency and high-frequency optical stimulation in kindling model, in which entorhinal stimulations were delivered ~4 s after the kindling stimulation (about the appearance of AD). Black rectangle indicates kindling stimulation, the green rectangle indicates a pulse of LFES, the blue rectangle indicates a pulse of blue optical stimulation, and the yellow rectangle indicates a trail of yellow optical stimulation. c) The experimental protocol for testing the effect of LFES in kindling model. In kindling acquisition experiments, we used 10 stimulations a day for three days in normal mice. In kindled seizure experiments, we used two or three stimulations a day for one to three days. Black rectangle indicates kindling stimulation while the blue rectangle indicates blue optical

stimulation. d) The experimental protocol for testing the effect of LFES in pilocarpine mouse model. That is, LFES (1 Hz) was delivered using a cycle of 15 min ‘on’, and 15 min ‘off’ for 4 h every day and EEG was recorded 8 h every day. Green rectangle indicates LFES.

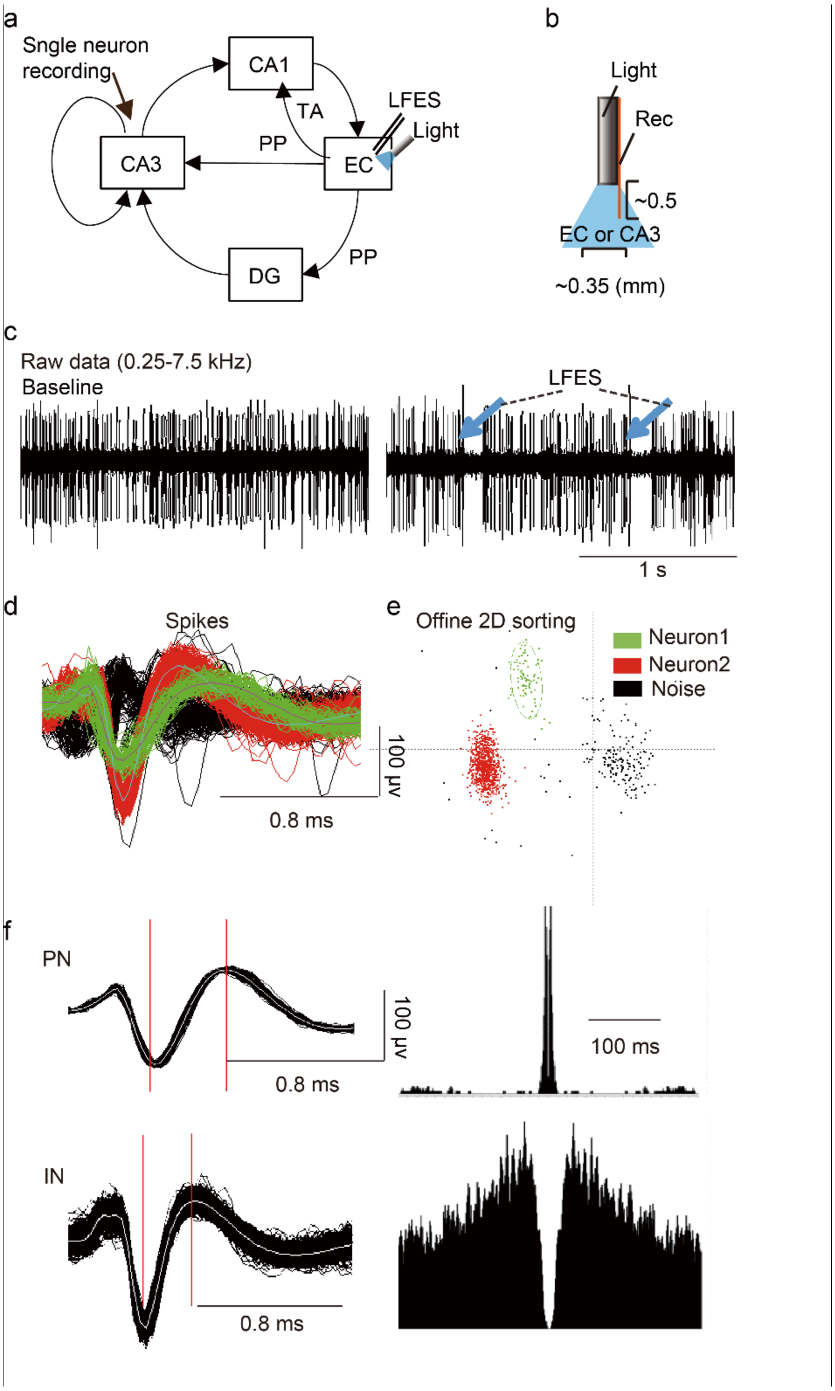

Fig.S2. Schematic of neuron recording and analysis. a) A Schematic of EC-hippocampal curits. b) The electrode design used for extracellular unit recordings during optical stimulation. c)

Recorded raw data (0.25-7 kHz) in the CA3. Left: baseline; Right: during entorhinal LFES. d) The picked waveform of recorded neurons. e) The 2-D clustered spike sorting. f) Classifying the recorded neurons according to their waveforms and autocorrelation patterns. The red line indicate the method for measurement of the spike widths.

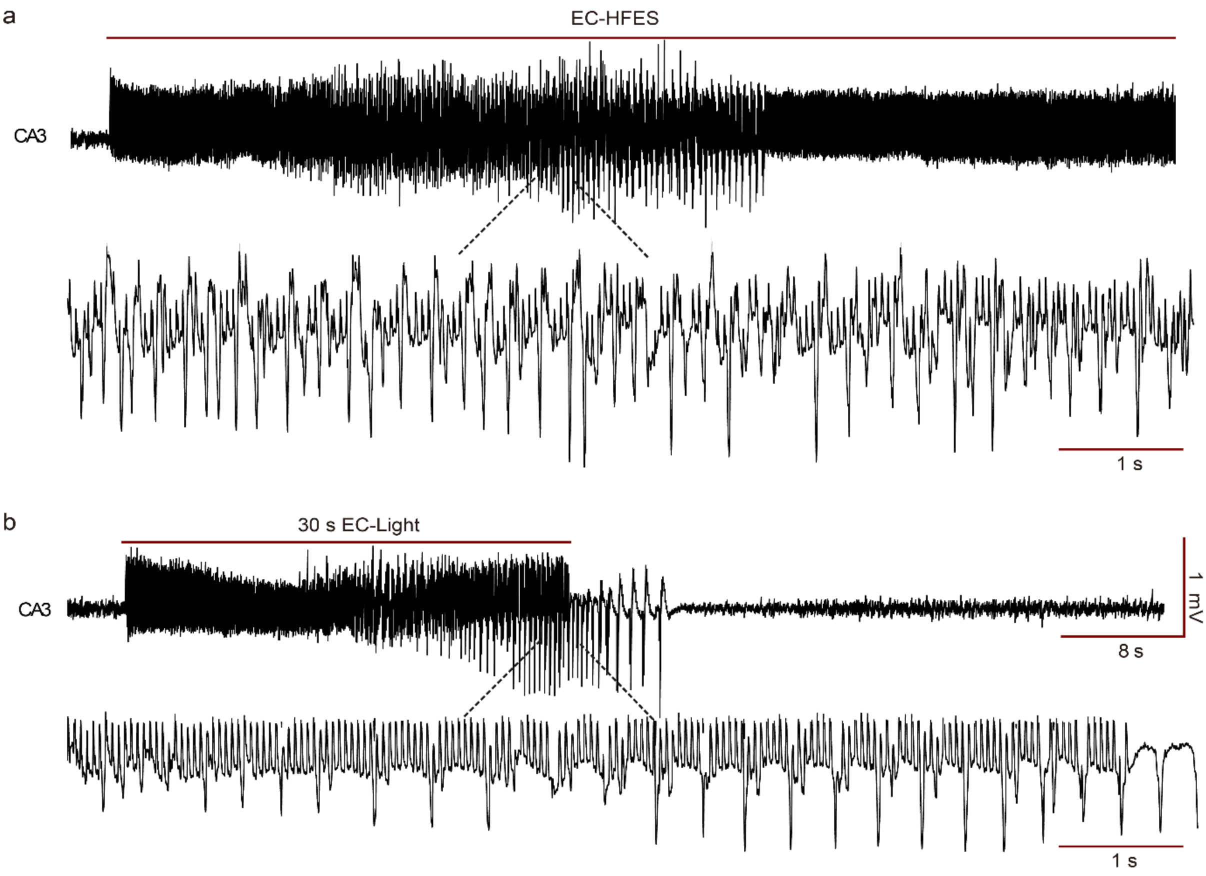

Fig. S3. Entorhinal HFES and high-frequency light activation of entorhinal PNs induce seizures. a) Entorhinal HFES induce seizure in a WT mice; b) high-frequency light activation of entorhinal PNs induce seizure in a *CaMKIIα-ChR2*^{EC} mice.

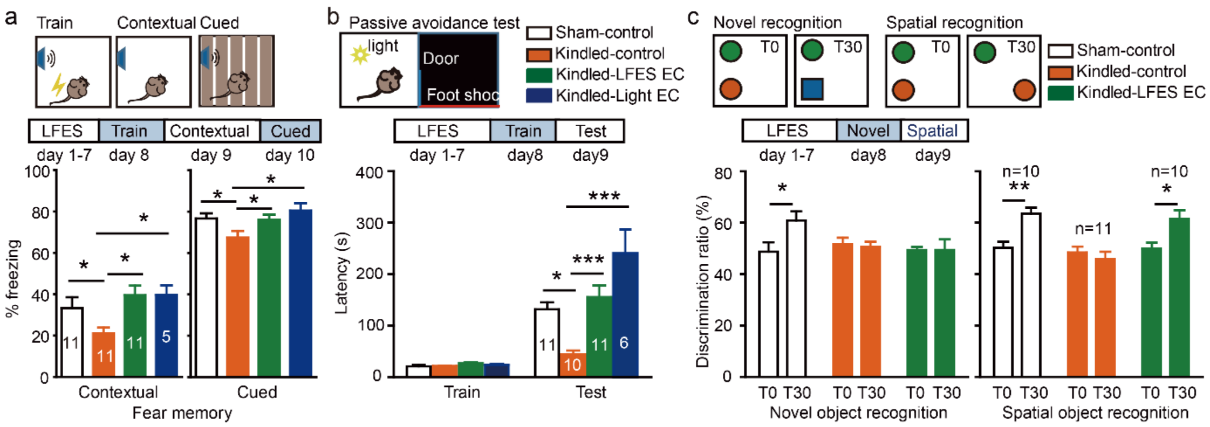

Fig. S4. Entorhinal LFES restores the impaired memory in kindled mice. a) The effects of entorhinal LFES or photo-stimulation on contextual and cued fear memory performance in kindled mice. *p < 0.05 compared to the kindled mice (one-way ANOVA followed by LSD *post hoc* test). The kindled-light EC group means selective activation of in entorhinal PNs in *CaMKIIα-ChR2*^{EC} mice by using photo-stimulation (1 Hz). b) The effects of entorhinal LFES or photo-stimulation on passive avoidance memory performance in kindled mice. *p < 0.05 and ***p < 0.001 compared to the kindled mice (one-way ANOVA followed by LSD *post hoc* test). c) The effects of entorhinal LFES on novel and spatial object recognition memory performance in kindled mice (T0, training session; T30, novel or spatial object recognition testing session). *p < 0.05 and **p < 0.01 compared to the each T0 training session (paired t-test). Different mice were used in the three behavioral test.

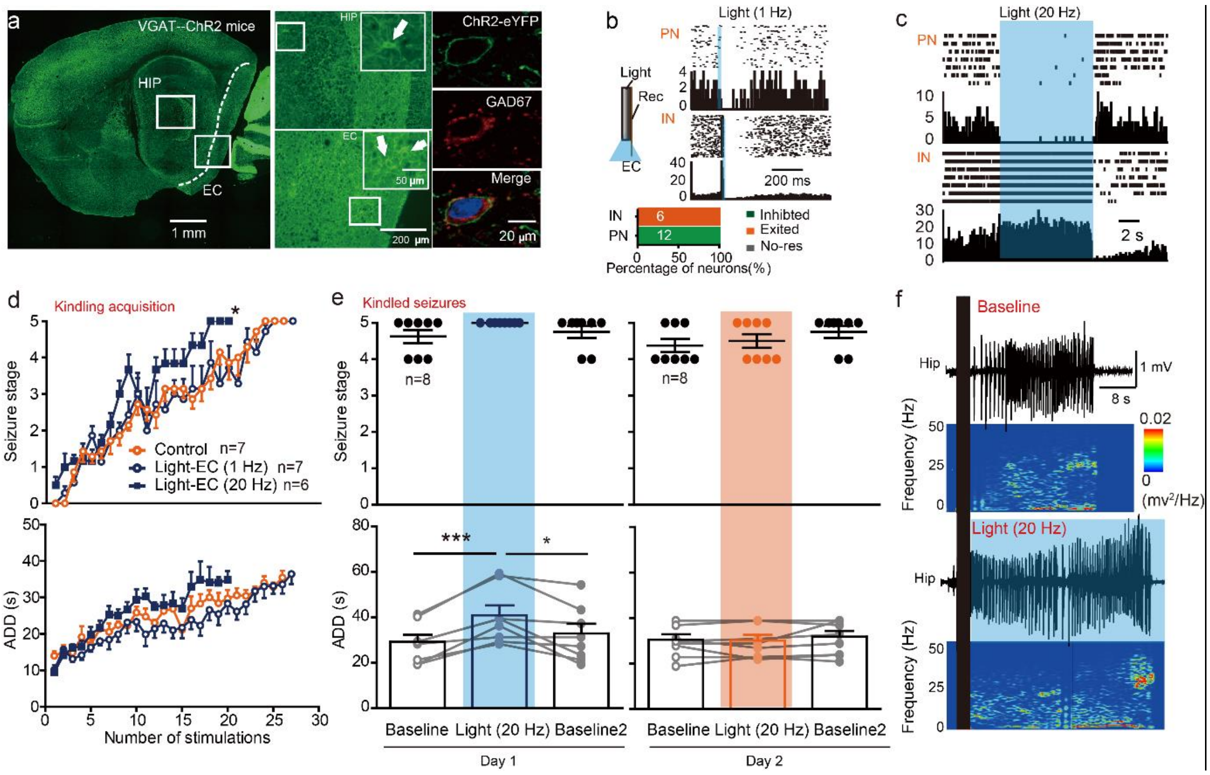

Fig. S5. Activation of entorhinal GABAergic neurons promotes hippocampal seizures. a) Immunohistochemical staining showing that ChR2-eYFP was expressed in GABAergic neurons in *VGAT-ChR2-eYFP* mice. b) Left: schematic of the electrode design for extracellular unit recording during blue light stimulation in the EC. Right: per-event rasters showing that blue light stimulation (473 nm, 1 Hz) excited INs like LFES. c) Blue light stimulation (20 Hz) continuously excited INs and inhibited PNs in the EC. d) Effect of entorhinal blue light stimulation (1 or 20 Hz) on behavioural seizure stage of (above) and ADD (below) during hippocampal kindling acquisition. *p <0.05 compared to control group (two-way ANOVA for repeated measures followed by LSD *post hoc* test). Control group was received yellow light stimulation (593 nm, 20 Hz). e) Effect of entorhinal blue light stimulation on seizure stage (above) and ADD (below) of kindled seizures. *p <0.05, ***p <0.001 compared to baseline

(paired *t* test). f) Representative EEGs showing that entorhinal blue light stimulation (20 Hz) prolonged the ADD; the black rectangle was indicated clearly as kindling stimulation.

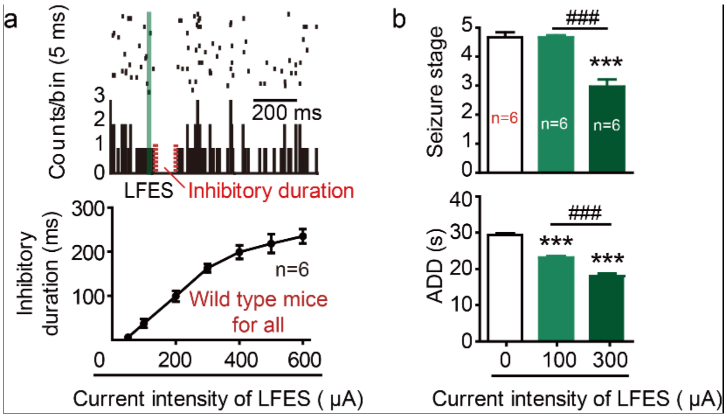

Fig. S6. The effect of current intensity on the inhibitory and antiepileptic effect of entorhinal LFES. a) The effect of current intensity on the inhibitory duration that was induced by entorhinal LFES in hippocampal neurons. b) The effect of current intensity on the antiepileptic effect of LFES in kindled mice. ***$p < 0.001$ compared to the control group and $^{\#\#\#}p < 0.001$ compared to the control 100 μA group (one-way ANOVA followed by LSD *post hoc* test).

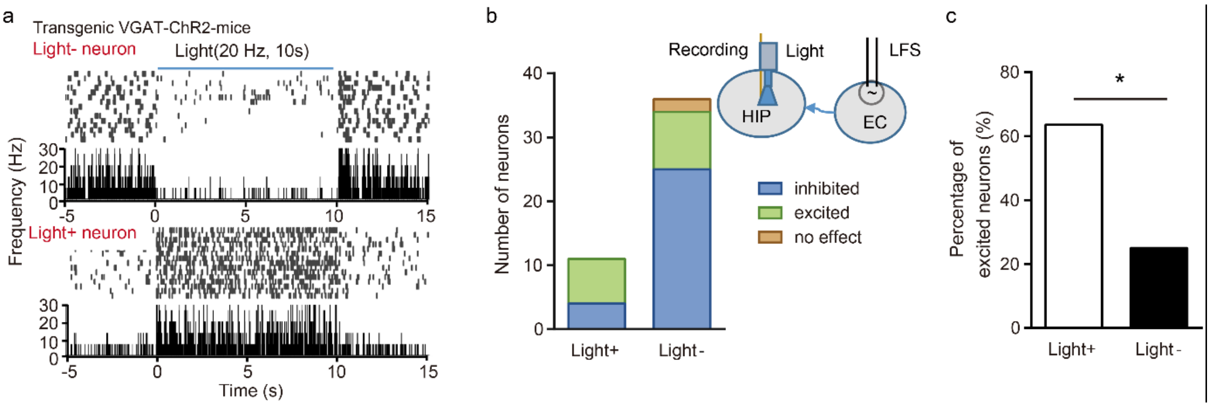

Fig. S7. The effect of entorhinal LFES on optogenetically identified hippocampal neurons in transgenic *VGAT-ChR2-eYFP* mice. a) Representative hippocampal neurons that were identified by their responses to light: PNs (upper, inhibited by light; Light-) and GABAergic neurons (lower, excited by light; Light+). Blue light, 200 pulses of 20 Hz blue light stimulation. b) The distribution of hippocampal PNs and GABAergic neurons according to their responses to LFS in the EC. c) The percentages of PNs and GABAergic neurons that were excited by entorhinal LFES (more percentage population of GABAergic neurons were excited). *$p < 0.05$ (χ^2 test).

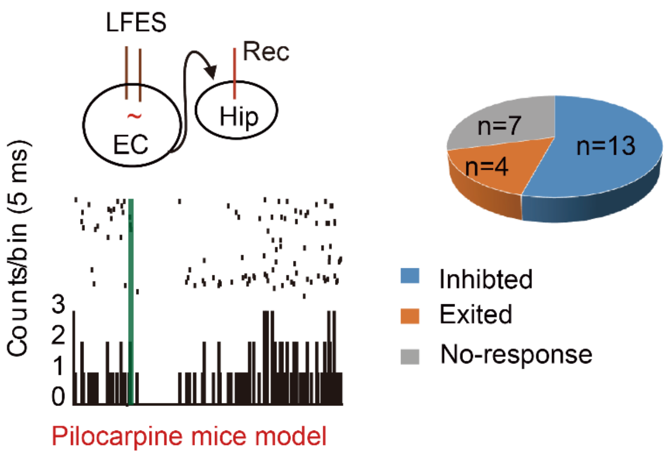

Fig. S8. Entorhinal LFES also had an inhibitory effect on PNs in mouse pilocarpine model of epilepsy. Left: Representative Per-event rasters; Right: statistical data.

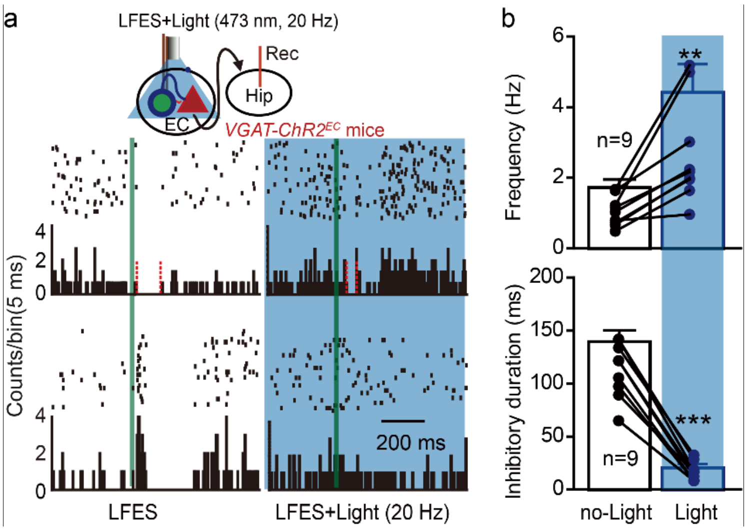

Fig. S9. Activation of entorhinal GABAergic neurons increases hippocampal neuronal activity and attenuated the inhibitory effect of LFES. a) Representative per-event rasters showing that entorhinal blue light stimulation (473 nm, 20 Hz, 5 ms per pulse) attenuated the inhibitory effect of entorhinal LFES in *VGAT-eChR2*^{EC} mice. Top plot: the effect of blue light stimulation on a PNs that inhibited LFES. Bottom plot: the effect of blue light stimulation on a PNs that excited LFES. b) Above: statistical data showing the effect of photo-stimulation on spiking frequency in hippocampal PNs. Below: statistical data showing the effect photo-stimulation on the inhibitory duration of hippocampal PNs that were induced by entorhinal LFES $^{**}p < 0.01$ compared to the baseline (paired *t*-test).

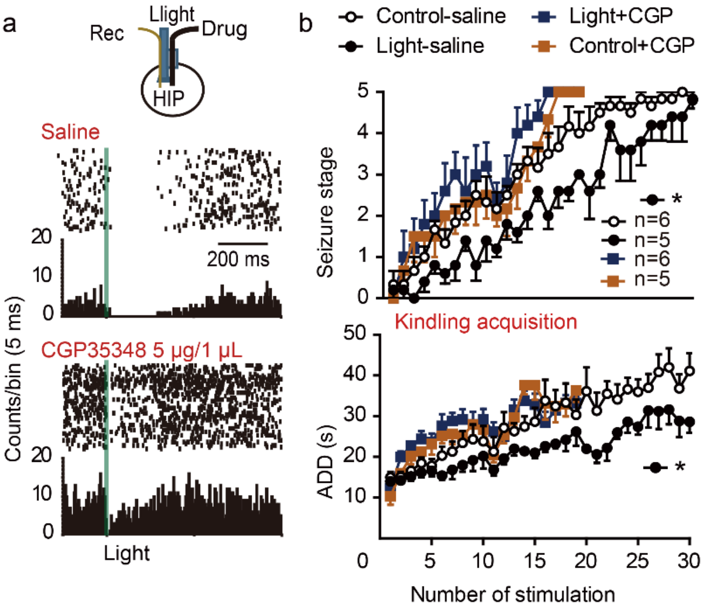

Fig. S10. GABA receptor antagonists attenuates both the inhibitory and the antiepileptic effects of focal light stimulation. a) Representative per-event rasters showing that injection of CGP35348 attenuated the inhibitory effect of focal photo-stimulation (473 nm, 1 Hz, 5 ms per pulse) on hippocampal neurons. Top plot: intrahippocampal injection of saline (1 μL). Bottom plot: Intrahippocampal injection of CGP35348 (5 μg in 1 μL). b) Intrahippocampal injection of CGP35348 attenuated the antiepileptic effect of focal photo-stimulation (473 nm, 1 Hz, 5 ms per pulse) during kindling acquisition. Control-saline and Light-saline: Intrahippocampal injection of saline (1 μL). Control-CGP and Light-CGP: Intrahippocampal injection of CGP35348 (5 μg in 1 μL). The injection of the drugs was performed twice per day, approximately every 2.5 h (during 5 kindling stimulations). *$p < 0.05$ compared to the saline group (two-way ANOVA for repeated measures).

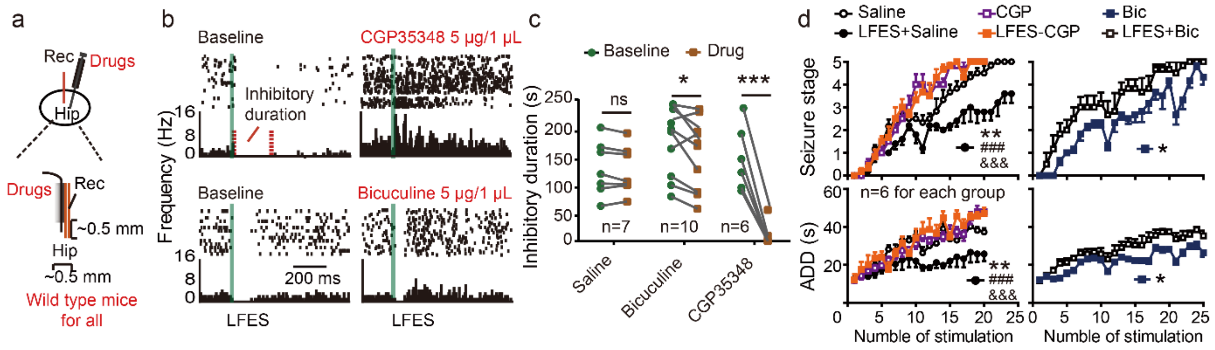

Fig. S11. GABA receptor antagonists attenuates both the inhibitory and the antiepileptic effects of entorhinal LFES. a) The strategy to investigate whether GABA receptor antagonists would affect the inhibitory and antiepileptic effects of entorhinal LFES. b-c) GABAergic receptor antagonist (bicuculline or CGP35348) that was injected into the hippocampus during recording attenuated the inhibitory effect of entorhinal LFES by reducing the duration of inhibition. b): representative per-event rasters; c): Statistical analysis. *$p < 0.05$ and ***$p < 0.001$ compared to the baseline (paired t-test). d) The effect of intrahippocampal injection of CGP35348 or bicuculline on the antiepileptic effect of entorhinal LFES in hippocampal kindling mice. *$p < 0.05$ and **$p < 0.001$ compared to the control group (two-way ANOVA for repeated measures followed by LSD *post hoc* test).

1 **Table S1. Details about statistical tests**

Figures	statistical object	method	df	t, F or χ2 value	P	*Post hoc* method
Fig. 1b	Seizure stage	Kruskal Wallis	3	χ2 = 20.470	<0.001	Mann-Whitney U
	ADD	Kruskal Wallis	3	χ2 = 15.269	0.002	Mann-Whitney U
Fig. 1c	Seizure stage	Two-way ANOVA	2	F = 16.950	<0.001	LSD
	ADD	Two-way ANOVA	2	F = 4.708	0.026	LSD
Fig. 1d	Seizure stage	Two-way ANOVA	1	F = 68.25	<0.001	None
	ADD	Two-way ANOVA	1	F = 24.61	<0.001	None
Fig. 1e	Seizure stage	Mann-Whitney U			0.002	None
	ADD	t test	10	t = 3.766	0.004	none
Fig. 1i	Ictal event (LFES *vs* LFES withheld)	Wilcoxon matched-pairs signed rank test			0.031	None
	Ictal event (control *vs* LFES)	Wilcoxon Signed Rank Test			0.031	None
	Ictal event (control *vs* LFES withheld)	Mann-Whitney U test			0.157	None
Fig. 1j	Interictal spike (LFES *vs* LFES withheld)	Paired t test	5	t = 6.574	0.001	None
	Interictal spike (control *vs* LFES)	t test	10	t = 3.177	0.010	None

	Interictal spike (LFES *vs* LFES withheld)	t test	10	t = 0.978	0.351	None
Fig. 2g	Seizure stage	Two-way ANOVA	3	F = 25.87	<0.001	LSD
	ADD	Two-way ANOVA	3	F = 6.623	0.002	LSD
Fig. 2h	Seizure stage	Two-way ANOVA	2	F = 14.027	<0.001	LSD
	ADD	Two-way ANOVA	2	F = 15.649	<0.001	LSD
Fig. 3d	PN firing rate	Paired t test	11	t = 5.977	<0.001	None
	IN firing rate	Paired t test	7	t = 2.103	0.074	None
Fig. 3f	Seizure stage	Two-way ANOVA	3	F = 38.558	<0.001	LSD
	ADD	Two-way ANOVA	3	F = 15.913	<0.001	LSD
Fig. 3g	Seizure stage	Kruskal Wallis	3	$\chi 2$ = 16.515	<0.001	Mann-Whitney U
	ADD	Kruskal Wallis	3	$\chi 2$ = 15.562	<0.001	Mann-Whitney U
Fig. 4c	PN firing rate	Paired t test	11	t = 5.121	<0.001	None
	IN firing rate	Paired t test	4	t = 4.092	0.015	None
Fig. 4d	Seizure stage	Two-way ANOVA	2	F = 10.816	<0.001	LSD
	ADD	Two-way ANOVA	2	F = 12.209	<0.001	LSD
Fig. 4e	Seizure stage	Kruskal Wallis	4	$\chi 2$ = 30.294	<0.001	Mann-Whitney U

	ADD	One-way ANOVA	4	F = 14.517	<0.001	LSD
Fig. 5d	Seizure stage	Two-way ANOVA	1	F = 44.089	<0.001	None
	ADD	Two-way ANOVA	1	F = 9.188	0.013	None
Fig. 5e	Seizure stage	Wilcoxon matched-pairs signed rank test			0.063	None
	ADD	Paired t test	6	t = 4.664	0.004	None
Fig. 6c	Number of excited IN	$\chi 2$	1	$\chi 2$ = 14.320	<0.001	None
	Number of excited IN	$\chi 2$	1	$\chi 2$ = 8.505	0.004	None
Fig. 6d	Number of excited IN	$\chi 2$	1	$\chi 2$ = 1.182	0.277	None
Fig. 6e	Inhibitory duration	Paired t test	7	t = 4.004	0.005	None
Fig. 6f	PN firing rate	Paired t test	13	t = 3.849	0.002	None
	IN firing rate	Paired t test	7	t = 1.738	0.126	None
Fig. 7c	Seizure stage	Kruskal Wallis	4	20.045	<0.001	Mann-Whitney U
	ADD	One-way ANOVA	4	F = 4.979	0.003	LSD
Fig. 7f	Percentage of excited neurons	$\chi 2$	1	$\chi 2$ = 11.03	0.001	None
Fig. S4a	Contextual fear memory	One-way ANOVA	3	F = 3.572	0.024	LSD
	Cured fear memory	One-way ANOVA	3	F = 3.315	0.031	LSD
Fig. S4b	Passive Avoidance (test)	One-way ANOVA	3	F = 12.100	<0.001	LSD

Fig. S4c	Novel recognition (Sham-Control)	Paired t test	9	t = 2.522	0.033	None
	Novel recognition (Kindled-LFES EC)	Paired t test	9	t = 0.128	0.901	None
	Spatial recognition (Sham-Control)	Paired t test	9	t = 4.076	0.003	None
	Spatial recognition (Kindled-LFES EC)	Paired t test	9	t = 3.022	0.014	None
Fig. S5d	Seizure stage	Two-way ANOVA	2	F = 4.087	0.036	LSD
	ADD	Two-way ANOVA	2	F = 4.554	0.026	LSD
Fig. S5e	Seizure stage (day 1) Baseline	Wilcoxon matched-pairs signed rank test			0.250	None
	Baseline2	Wilcoxon matched-pairs signed rank test			0.500	None
	ADD (day 1) Baseline	Paired t test	7	t = 5.921	<0.001	None
	Baseline2	Paired t test	7	t = 3.147	0.016	None
	Seizure stage (day 2) Baseline	Wilcoxon matched-pairs signed rank test			> 0.999	None
	Baseline2	Wilcoxon matched-pairs signed rank test			0.500	None
	ADD (day 2)	Paired t test	7	t = 0.331	0.750	None

	Baseline					
	Baseline2	Paired t test	7	t = 1.217	0.263	None
Fig. S6b	Seizure stage	Kruskal Wallis	2	χ2 = 10.451	<0.001	Mann-Whitney U
	ADD	One-way ANOVA	2	F = 105.300	<0.001	LSD
Fig. S7c	Percentage of excited neurons	χ2	1	χ2 = 5.601	0.018	None
Fig. S8b	Frequency	Paired t test	8	t = 4.122	0.003	None
	Inhibitory duration	Paired t test	8	t = 13.93	<0.001	None
Fig. S10b	Seizure stage	Two-way ANOVA	3	F = 6.435	0.004	LSD
	ADD	Two-way ANOVA	3	F = 3.797	0.029	LSD
Fig. S11c	Inhibitory duration (saline)	Paired t test	6	t = 1.044	0.337	None
	Inhibitory duration (bicuculline)	Paired t test	9	t = 2.438	0.038	None
	Inhibitory duration (CGP35348)	Paired t test	5	t = 8.49	<0.001	None
Fig. S11d	Seizure stage (left)	Two-way ANOVA	3	F = 14.679	<0.001	LSD
	ADD (left)	Two-way ANOVA	3	F = 6.798	0.003	LSD
	Seizure stage (right)	Two-way ANOVA	1	F = 7.53	0.021	None
	ADD (right)	Two-way ANOVA	1	F = 9.906	0.010	None

References

Alexander, G.M., Rogan, S.C., Abbas, A.I., Armbruster, B.N., Pei, Y., Allen, J.A., Nonneman, R.J., Hartmann, J., Moy, S.S., Nicolelis, M.A., McNamara, J.O., Roth, B.L, 2009. Remote control of neuronal activity in transgenic mice expressing evolved G protein-coupled receptors. Neuron *63*, 27-39.

Favrais, G., van de Looij, Y., Fleiss, B., Ramanantsoa, N., Bonnin, P., Stoltenburg-Didinger, G., Lacaud, A., Saliba, E., Dammann, O., Gallego, J., *et al.*, 2011. Systemic inflammation disrupts the developmental program of white matter. Annals of neurology *70*, 550-565.

King, S.L., Marks, M.J., Grady, S.R., Caldarone, B.J., Koren, A.O., Mukhin, A.G., Collins, A.C., and Picciotto, M.R., 2003. Conditional expression in corticothalamic efferents reveals a developmental role for nicotinic acetylcholine receptors in modulation of passive avoidance behavior. The Journal of neuroscience: the official journal of the Society for Neuroscience *23*, 3837-3843.

Kinnavane, L., Amin, E., Olarte-Sánchez, C.M., Aggleton, J.P., 2016. Detecting and discriminating novel objects: The impact of perirhinal cortex disconnection on hippocampal activity patterns. Hippocampus. 26:1393-1413

Liu, L., Zhang, S., Zhu, Y., Fu, Q., Zhu, Y., Gong, Y., Ohtsu, H., Luo, J., Wei, E., and Chen, Z., 2007. Improved learning and memory of contextual fear conditioning and hippocampal CA1 long-term potentiation in histidine decarboxylase knock-out mice. Hippocampus *17*, 634-641.

Paxinos, G., and Franklin, K., 2001. The Mouse Brain in Stereotaxic Coordinates, Second

Edition edn (San Diego: Academic Press).

Paxinos, G., and Watson, C., eds., 2004. The Rat Brain in Stereotaxic Coordinates (New York: Academic Press).

Tonnesen, J., Sorensen, A.T., Deisseroth, K., Lundberg, C., and Kokaia, M, 2009. Optogenetic control of epileptiform activity. Proceedings of the National Academy of Sciences of the United States of America *106*, 12162-12167.

Wang, Y., Liang, J., Xu, C., Wang, Y., Kuang, Y., Xu, Z., Guo, Y., Wang, S., Gao, F., and Chen, Z., 2016. Low-frequency stimulation in anterior nucleus of thalamus alleviates kainate-induced chronic epilepsy and modulates the hippocampal EEG rhythm. Experimental neurology *276*, 22-30.
